# Variation in herbivore space use: comparing two savanna ecosystems with different anthrax outbreak patterns in southern Africa

**DOI:** 10.1186/s40462-023-00385-2

**Published:** 2023-07-31

**Authors:** Yen-Hua Huang, Norman Owen-Smith, Michelle D. Henley, J. Werner Kilian, Pauline L. Kamath, Sunday O. Ochai, Henriette van Heerden, John K. E. Mfune, Wayne M. Getz, Wendy C. Turner

**Affiliations:** 1 Wisconsin Cooperative Wildlife Research Unit, Department of Forest and Wildlife Ecology, University of Wisconsin-Madison, Madison, WI 53706 USA; 2 Centre for African Ecology, School of Animal, Plant and Environmental Sciences, University of the Witwatersrand, Wits, 2050 South Africa; 3 Applied Behavioural Ecology and Ecosystem Research Unit, School of Environmental Sciences, University of South Africa, Florida, Johannesburg, 1710 South Africa; 4 Elephants Alive, Ekuthuleni Shareblock Ltd, Hoedspruit, 1380 South Africa; 5 Department of Philosophy, Faculty of Humanities, University of Johannesburg, Auckland Park, 2006 South Africa; 6 Etosha Ecological Institute (retired), Etosha National Park, Ministry of Environment, Forestry and Tourism, Okaukuejo, Namibia; 7 School of Food and Agriculture, University of Maine, Orono, ME 04469 USA; 8 Department of Veterinary Tropical Diseases, University of Pretoria, Onderstepoort, South Africa; 9 Department of Environmental Science, University of Namibia, Windhoek, Namibia; 10 Department of Environmental Science, Policy & Management, University of California, Berkeley, CA 94704 USA; 11 School of Mathematical Sciences, University of KwaZulu-Natal, Durban, South Africa; 12 Wisconsin Cooperative Wildlife Research Unit, U.S. Geological Survey, Department of Forest and Wildlife Ecology, University of Wisconsin-Madison, Madison, WI 53706 USA

**Keywords:** *Aepyceros melampus*, *Antidorcas marsupialis*, *Bacillus anthracis*, *Connochaetes taurinus*, Disease transmission, *Equus quagga*, Home range, *Loxodonta africana*, *Syncerus caffer*, *Tragelaphus strepsiceros*

## Abstract

**Background:**

The distribution of resources can affect animal range sizes, which in turn may alter infectious disease dynamics in heterogenous environments. The risk of pathogen exposure or the spatial extent of outbreaks may vary with host range size. This study examined the range sizes of herbivorous anthrax host species in two ecosystems and relationships between spatial movement behavior and patterns of disease outbreaks for a multi-host environmentally transmitted pathogen.

**Methods:**

We examined range sizes for seven host species and the spatial extent of anthrax outbreaks in Etosha National Park, Namibia and Kruger National Park, South Africa, where the main host species and outbreak sizes differ. We evaluated host range sizes using the local convex hull method at different temporal scales, within-individual temporal range overlap, and relationships between ranging behavior and species contributions to anthrax cases in each park. We estimated the spatial extent of annual anthrax mortalities and evaluated whether the extent was correlated with case numbers of a given host species.

**Results:**

Range size differences among species were not linearly related to anthrax case numbers. In Kruger the main host species had small range sizes and high range overlap, which may heighten exposure when outbreaks occur within their ranges. However, different patterns were observed in Etosha, where the main host species had large range sizes and relatively little overlap. The spatial extent of anthrax mortalities was similar between parks but less variable in Etosha than Kruger. In Kruger outbreaks varied from small local clusters to large areas and the spatial extent correlated with case numbers and species affected. Secondary host species contributed relatively few cases to outbreaks; however, for these species with large range sizes, case numbers positively correlated with outbreak extent.

**Conclusions:**

Our results provide new information on the spatiotemporal structuring of ranging movements of anthrax host species in two ecosystems. The results linking anthrax dynamics to host space use are correlative, yet suggest that, though partial and proximate, host range size and overlap may be contributing factors in outbreak characteristics for environmentally transmitted pathogens.

**Supplementary Information:**

The online version contains supplementary material available at 10.1186/s40462-023-00385-2.

## Introduction

Infectious disease dynamics are influenced by the movements of animal hosts [1, 2]. Different host movement patterns can alter contact networks among individuals, affecting transmission dynamics of directly transmitted diseases [3]. Moreover, animal hosts using a heterogeneous landscape have different exposure risk to environmentally transmitted pathogens, based on habitat types and landscape features [4, 5]. As a result, an understanding of animal movement ecology is an important foundation to better understand disease dynamics.

The size of the area used is an important characteristic in animal movement studies [6], and can be influenced by various factors, including age, sex, reproductive status, habitat, resource availability, diet and body size [7–13]. The area used by an individual is often loosely referred to as its “home range” [14], implying a defined area is used [15]. However, site fidelity—the tendency to utilize the same area [16]—varies across species and individuals, and among mammals, ungulates often have low site fidelity [17]. Since this study focuses on ungulate herbivores, we use the term “range size” instead of home range, throughout. Movements of ungulate herbivores may be nomadic, searching for resources across large ranges with few revisitations, especially in unpredictable or resource-poor environments [18]; though within these relatively nomadic species, individuals may be situationally territorial, occupying relatively small ranges, such as males around conception periods [19].

Comparing among and within species, larger host range size has been linked with higher parasite richness or diversity across a variety of host taxa [20–26]. This positive correlation may be due to increased pathogen transmission when larger range size increases the probability of contacting more infectious individuals or areas [27]. However, smaller range sizes may also heighten transmission of environmentally transmitted parasites due to repeated use of the same high-risk areas. For example, territorial male Grant’s gazelle (*Nanger granti*) and Thomson’s gazelle (*Gazella thomsoni*) utilize smaller ranges than their conspecifics without territories and have higher intensities of gastrointestinal parasite infections [28]. Thus, range size may be expected to influence disease transmission, but more research could help understand broad patterns in relationships between range sizes and infections for a variety of host and parasite taxa.

This study examines host range size patterns in the context of the disease anthrax. Anthrax is a multi-host, highly lethal and acute disease that kills infected hosts within a week of exposure [29]. This environmentally transmitted disease infects mainly herbivorous mammals and is caused by the bacterial pathogen *Bacillus anthracis*. Anthrax transmission relies upon host exposure to spores present in environmental reservoirs such as anthrax carcass sites [30, 31] (with biotic vectors contributing to cases in some systems [32, 33]). Though water can be considered a transmission source for *B. anthracis* [34], point water sources are unlikely to be transmission reservoirs [30]. While environmental factors and host behavioral traits have been associated with anthrax risk in a variety of ecosystems across the pathogen’s global range, these are often quite different from one ecosystem to another, making general patterns of risk difficult to discern [35–37].

**Fig. 1:**
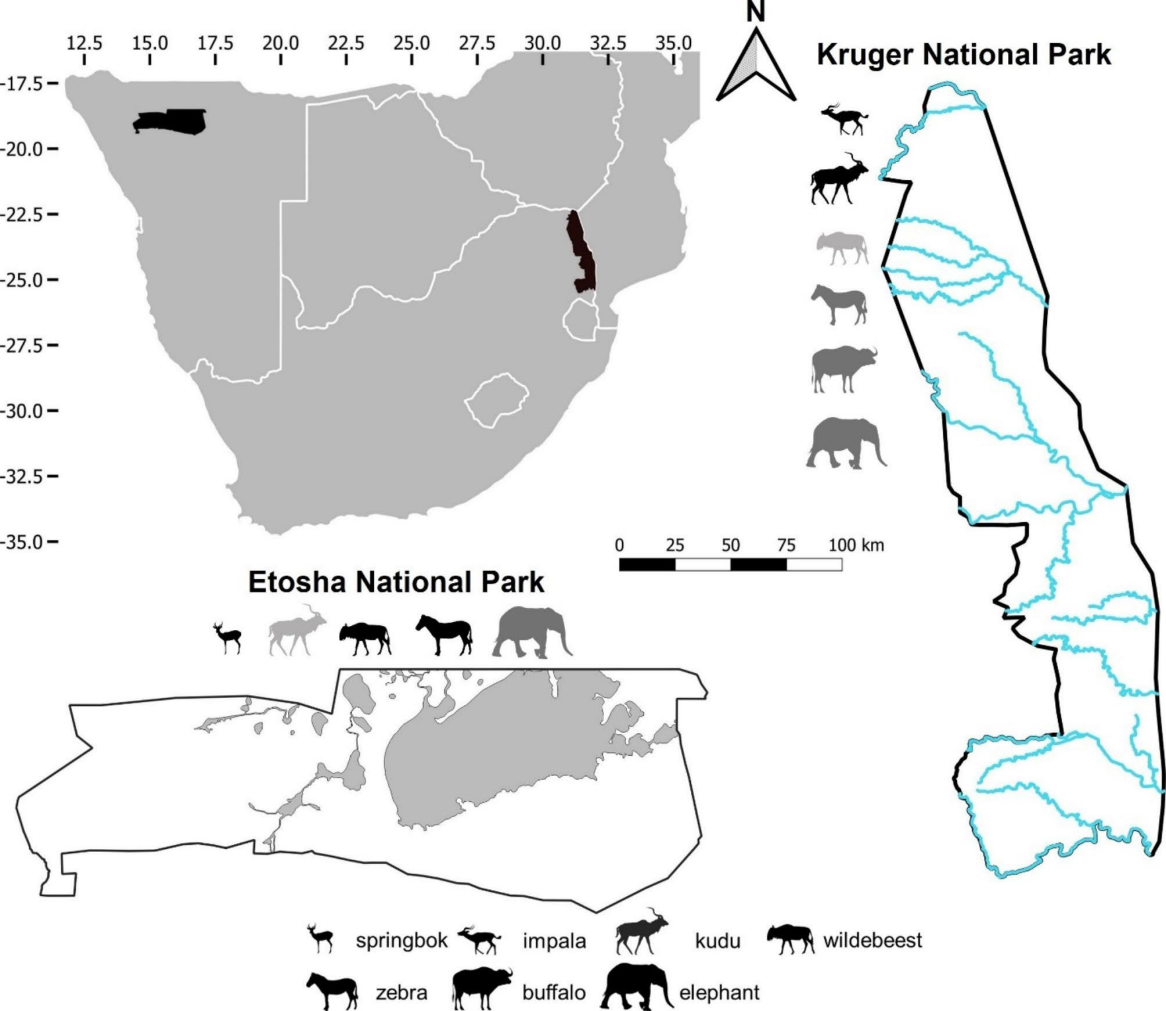
The study areas Etosha National Park, Namibia and Kruger National Park, South Africa in southern Africa. Animal silhouettes represent study species in the parks, including springbok (*Antidorcas marsupialis*) in Etosha, impala (*Aepyceros melampus*) and African buffalo (*Syncerus caffer*) in Kruger, and greater kudu (*Tragelaphus strepsiceros*), blue wildebeest (*Connochaetes taurinus*), plains zebra (*Equus quagga*) and African elephant (*Loxodonta africana*) in both parks, with buffalo absent in Etosha and springbok in Kruger. Wildebeest is more rarely found in far north of Kruger, and we did not have movement data on impala in Etosha. Host species comprising > 12% of anthrax cases in each park (1976–2014 for Etosha and 2010–2015 for Kruger) are in black; between 12% and 4% are in dark grey; and < 4% are in light grey. The grey areas in Etosha and blue lines in Kruger are salt pans and perennial rivers, respectively which are potential boundaries for animal movements. The scale bar is related to the maps of both parks. The numbers framing southern Africa indicate degrees of latitude and longitude

Anthrax is endemic in both Etosha National Park, Namibia and Kruger National Park, South Africa (Fig. 1) [35]. Potential host species in these two parks include springbok (*Antidorcas marsupialis*), impala (*Aepyceros melampus*), greater kudu (*Tragelaphus strepsiceros*), blue wildebeest (*Connochaetes taurinus*), plains zebra (*Equus quagga*), African buffalo (*Syncerus caffer*) and African elephant (*Loxodonta africana*) [35], with buffalo absent in Etosha and springbok absent in Kruger. Both parks have semi-arid African savanna ecosystems and share many animal species that are also potential anthrax hosts. However, Kruger has higher water availability and vegetation productivity than Etosha [35, 38, 39], and the two parks have very different patterns in anthrax infections [35]. Outbreaks in Etosha occur annually with typically 10–100 anthrax mortalities detected in an outbreak [35]. In contrast, sporadic large outbreaks in Kruger can impact 100–1000 herbivorous mammals, occurring every 10–20 years [35]. Further, the most commonly infected species in Etosha is zebra, followed by springbok, wildebeest and elephant, while there are rarely anthrax cases in kudu and impala [4, 35]. In contrast, kudu and impala are the main host species in Kruger followed by buffalo, whereas zebra and elephant have relatively few cases, and wildebeest rarely contribute to anthrax outbreaks [35].

Animal behavior is likely an important factor affecting anthrax transmission [35, 40, 41], for example, zebra habitat selection and diet selection drive anthrax dynamics in Etosha [4, 42]. However, anthrax dynamics are also driven by more complex mechanisms [35] which possibly involves interactions between hosts and the environment, food-web feedbacks [43], and biotic vectors [32, 44], or other unknown driving factors. Host individuals may need to have multiple contacts to contract the disease [45–47], and species with small range size have been suggested to have heightened anthrax exposure [44], but no study has yet investigated this connection. Apart from a potential change in exposure risk with different range sizes, the large range sizes of high mobility species may also contribute to the spatial spread of an outbreak across a landscape [48, 49]. Despite the expectation that a sick animal might move less than a healthy animal, the peracute to acute nature of this disease may preclude a period of sickness behavior prior to death. As an example, movement trajectory indices for hippopotamus (*Hippopotamus amphibius*) in Tanzania did not differ before and after anthrax infection [50]. Movements of infected animals using large ranges may thus translocate *B. anthracis* beyond the initial outbreak area, extending the spatial extent of an anthrax outbreak [44, 50]. Because of the potential effects of range size on anthrax transmission, this study hence examines the range size of multiple host species to explore the relationship between host range size and anthrax dynamics.

Our objectives were to investigate (1) if range size and within-individual range overlap affected anthrax risk, and (2) if outbreak spatial extent was associated with high mobility host species. We first estimated range sizes for seven potentially common anthrax host species at three different temporal scales, using the local convex hull (LoCoH) method [51, 52]. We compared temporal heterogeneity in animal space use among species, and evaluated whether range size differed with species and park, and whether more commonly infected species in each park utilized smaller ranges. We further investigated within-individual range overlap from one month to the next as an indication for potential risk of repeated anthrax exposures, to evaluate whether species having more anthrax cases also had higher range overlap. We then investigated the spatial extent of anthrax mortalities in each park from decades of anthrax surveillance data. We compared outbreak spatial extent with factors including total case numbers, number of species affected, and case numbers in common host species in an outbreak, to evaluate potential species contribution to outbreak extent. Sampling periods for the movement data varied with species and parks, preventing us from directly comparing anthrax outbreaks with contemporaneous host space use. However, the main host species in the two parks remained very similar over years [35], providing an opportunity to examine the associations with basic animal movement ecology. This study helps us advance our understanding of variation in anthrax transmission and the potential link with host space use across systems.

## Methods

### Study areas

Data for this study were collected in two national parks in southern Africa, Etosha and Kruger (Fig. 1), where anthrax primarily affects wild herbivores. In both parks anthrax is considered an endemic disease, contributing to seasonal and annual herbivore mortality patterns, with minimal interventions to reduce disease spread. Etosha is a semi-arid savanna (average annual rainfall in the central Etosha: 358 mm [53]), with three seasons: wet season in January – April, dry (early-dry) season in May – August, and semi-dry (late-dry) season in September – December. Rainfall is strongly seasonal and occurs mainly between November and April, with the greatest monthly rainfall occurring in January and February [54]. Animals rely on seasonal water from rainfall, or perennial water at boreholes, artesian or contact springs [55]. Much of Etosha is covered by mopane (*Colophospermum mopane*) shrubveld or treeveld, and open grasslands along a large salt pan. Vegetation in Kruger is characterized by woody, shrubland and open savannas [56], with higher canopy cover than Etosha. Kruger also has higher water availability than Etosha (average annual rainfall in the far north of Kruger: 430 mm [57]), from seasonal water and perennial boreholes, dams, springs, pools, and rivers flowing west-east [58]. In Kruger, the seasons based on rainfall occur one month earlier than Etosha: wet season in December – March, early-dry season in April – July, and late-dry season in August – November [59]. Unlike Etosha, there is still occasional rainfall during the dry period in Kruger [56, 60].

### Animal telemetry data

The study species considered here are the potential anthrax host species in the two parks, including springbok, impala, kudu, blue wildebeest, zebra, buffalo and elephant (Fig. 1) [35]. Although contributions to anthrax outbreaks vary with species and park (Table 1) [35], this group of species represents the majority of anthrax cases observed in the two parks. We compiled movement data from GPS (Global Positioning System) collars including newly collected and previously published datasets on springbok from Etosha, common impala (*A. m. melampus*) and buffalo from Kruger, and kudu, wildebeest, zebra and elephant from both parks between 2006 and 2020 (numbers, time periods and data sources in Table 2). Springbok and buffalo are only found in one of these parks; there are black-faced impala (*A. m. petersi*) in Etosha, but no movement data were available for this species. These tracked individuals in Etosha often utilized the anthrax high incidence region (central Etosha; Additional file 3: Figure S1, S2 and S3) [46]; however, in Kruger, only tracked impala, kudu and elephant stayed in or crossed the highest anthrax incidence region in the far north of the park (Pafuri), whereas buffalo, zebra and wildebeest were not tracked within the high-risk area (Additional file 3: Figure S1, S2 and S3) [46] due to regionally restricted space use and limited data availability. Tracked individuals of kudu in Etosha and wildebeest, zebra and buffalo in Kruger were restricted to only adult females, other species included adult males and females (Table 2).

**Table 1:** Opportunistically observed species contributions to anthrax cases and species main anthrax seasons in Etosha National Park, Namibia and Kruger National Park, South Africa for study species. Anthrax mortality from central Etosha 1976–2014 and northern Kruger 1990–2015 were retrieved from published data used in Huang et al. [35]. Because host compositions in Kruger varied temporally, this table shows the species contributions for the entire period and the period with a recent outbreak (2010–2015). Species contributions are likely biased against smaller species, and these species are ordered based on increasing body mass (Additional file 2: Table S5). Though wildebeest may be affected by anthrax in Kruger, they are rarely present at the highest incidence region in the park (Pafuri)

species	contribution to anthrax cases in Etosha 1976–2014 (%)	contribution to anthrax cases in Kruger 1990–2015 (%)	contribution to anthrax cases in Kruger 2010–2015 (%)	anthrax season
springbok (*Antidorcas marsupialis*)	17.3	not applicable	not applicable	wet
impala (*Aepyceros melampus*)	< 3.0	22.4	52.2	wet
greater kudu (*Tragelaphus strepsiceros*)	< 3.0	36.6	13.1	late dry
blue wildebeest (*Connochaetes taurinus*)	15.5	< 4.0	< 4.0	wet
plains zebra (*Equus quagga*)	54.4	2.9	5.2	wet
African buffalo (*Syncerus caffer*)	not applicable	23.4	11.0	late dry
African elephant (*Loxodonta africana*)	9.8	1.8	4.5	late dry

**Table 2:** Summary of numbers of individuals and tracking periods of herbivorous anthrax host species in Etosha National Park, Namibia and Kruger National Park, South Africa

species	number of males	number of females	tracking period	reference
	Etosha National Park			
springbok (*Antidorcas marsupialis*)	7	5	August 2009 – December 2010	[80, 81]
greater kudu (*Tragelaphus strepsiceros*)	0	10	July 2019 – November 2020	this study
blue wildebeest (*Connochaetes taurinus*)	18	16	July 2018 – October 2020	this study
plains zebra (*Equus quagga*)	13	24	April 2009 – December 2010 (9 individuals); August 2018 – October 2020 (28 individuals)	[4, 82]
African elephant (*Loxodonta africana*)	12	22	November 2008 – March 2015	[83, 84]
	Kruger National Park			
impala (*Aepyceros melampus*)	13	10	October 2018 – April 2020	this study
greater kudu (*Tragelaphus strepsiceros*)	12	15	October 2018 – September 2020	this study
blue wildebeest (*Connochaetes taurinus*)	0	10	April 2009 – March 2012	[85–88]
African buffalo (*Syncerus caffer*)	0	9	June 2005 – April 2013	[89, 90]
plains zebra (*Equus quagga*)	0	9	May 2006 – March 2012	[85, 88–91]
African elephant (*Loxodonta africana*)	6	6	July 2009 – November 2017	[92]

Because of different sampling intensities and irregular intervals of the telemetry data, we thinned the data to three readings a day for more comparable relocation data across different species and tracking periods among species. We divided days into morning (6:00–12:00; GMT + 1 for Etosha and GMT + 2 for Kruger), afternoon (12:00–18:00) and night (18:00–6:00), and extracted readings closest to 9:00, 15:00 and 24:00 for the three periods of a day for each individual (following the same procedures as Huang et al. [4]). We then prepared three different datasets for estimation of range size at bimonthly, monthly and seasonal scales. The bimonthly scale has two intervals per month: days 1–15 and day 16 to the month’s end. After a lethal exposure, herbivores are likely to die of anthrax within a few days to a week [29, 61]. Thus, a bimonthly interval is an appropriate scale for analyses in regard to anthrax risk. However, due to the low intensity of readings, we were limited to use longer intervals when comparing temporal heterogeneity. For the preparation of the datasets, we removed a time interval from an individual if its readings were fewer than two-thirds of the total possible readings of the interval (i.e., fewer than 30, 60 and 240 for bimonthly, monthly and seasonal intervals, respectively). Because of the inclusion criteria, the numbers of individuals as well as sample sizes varied among datasets.

### Range size and overlap

We used the three temporal datasets (at bimonthly, monthly and seasonal scales) to estimate 95% range sizes at the corresponding temporal scales. Comparing range sizes across temporal scales may provide information on temporal heterogeneity in animal space use. For example, if range sizes are similar across temporal scales, an individual may utilize a resident range and rarely show nomadic behavior. We calculated 95% ranges using the LoCoH, because of the potential boundaries of animal spatial distribution in the two parks, such as salt pans, rivers and fence lines [51, 52]. To estimate range sizes, we used *a*-LoCoH (adaptive local convex hull), with parameter *a* equal to maximum distance between two readings in the interval, since this *a* value is close to optimal *a* value for range estimation [51]. Moreover, we excluded individuals with fewer than three different seasons of data from the seasonal dataset, to provide longitudinal aspects of movements, and used this dataset to estimate range size and net squared displacement (NSD). NSD measures squared distances between relocations and a starting location [62], and its time-series provide information on animal trajectories [63]. To examine whether large range sizes can be linked with long traveling distances, NSD was calculated for each individual starting from the first time point of the data (Additional file 1: Supplementary Methods). We evaluated whether range sizes varied with resource availability using a remotely sensed index of vegetation greenness and biomass, Normalized Difference Vegetation Index (NDVI), to assess resource availability. We extracted average NDVI values in seasonal 95% ranges and tested whether seasonal range size variation between the two parks could be described by species identity and resource availability (Additional file 1: Supplementary Methods).

We used the monthly dataset to estimate within-individual range overlap from one month to the next, by calculating the average proportion of an individual’s monthly 95% range which was intersected by its range from the previous month (between zero and one) [64]. A high proportion of overlap implies an individual repeatedly visits the same areas which were utilized in the previous month. We evaluated range overlap at the monthly scale to have more readings to more accurately estimate the overlap. We excluded individuals with fewer than six pairs of consecutive months from the monthly dataset for range overlap estimation.

We tested the hypothesis that anthrax risk varies with range size by examining whether range size or range overlap drove species anthrax incidence. We fit species contribution to anthrax cases in each park (Table 1) to either species monthly average range size or range overlap using linear regressions, despite small sample sizes (*N* = 5 species for Etosha; *N* = 6 species for Kruger). Range sizes were square root transformed before fitting into the regressions due to their skewness.

### Spatial extent of anthrax mortalities

We investigated the spatial extent of anthrax mortality distribution by year, comparing the two parks, and evaluated the effect of host species on the distribution of anthrax cases. Since animal mortality surveillance in both parks is opportunistic, biases likely exist against recording anthrax deaths in smaller than larger species. In this study, anthrax mortality included anthrax confirmed cases from blood smear examination, bacterial culture, or molecular diagnosis from blood swabs, as well as anthrax suspected cases diagnosed by symptoms (i.e., blood exudation) [29] in cases where no samples were collected. We obtained data on coordinates of individual anthrax mortality events from 1996 to 2014 in Etosha and from 1990 to 2015 in Kruger through the Etosha Ecological Institute and Office of the State Veterinarian in Kruger, respectively. We used rainfall years from July to June (e.g., July 2006–June 2007 is the 2007 rainfall year) for both parks, to capture most outbreaks occurring during these time periods.

We estimated spatial extent of annual anthrax mortalities. Although surveillance effort may vary with years and regions, the mortality datasets can still provide useful estimates of the spatial extent of the outbreaks. We first removed years with fewer than ten anthrax mortalities with coordinates, to have enough cases to estimate ranges. We then calculated a 50% and 95% spatial extent of anthrax mortalities using the LoCoH. To estimate extent, we used *a*-LoCoH (adaptive local convex hull), with parameter *a* equal to maximum distance between two mortalities in the same year. We evaluated whether spatial extent was related to number of cases, number of species involved, and number of cases in common host species in each park. Common host species here included springbok, blue wildebeest, plains zebra and African elephant in Etosha, and impala, greater kudu, zebra, African buffalo and elephant in Kruger (Table 1) [35]. Associations of spatial extent and with other factors were evaluated with linear regressions with only one predictor in a model due to small sample sizes (*N* = 16 for Etosha; *N* = 13 for Kruger).

All of the analyses in this study were done using R v. 4.1.2 [65]. LoCoH and range overlap calculations were performed using package amt [66], and linear regressions were performed using package stats [65]. NDVI was downloaded from the National Aeronautics and Space Administration (NASA) Land Processes Distributed Active Archive Center by package MODIStsp [67], and processed by packages raster [68] and exactextractr [69]. Spatial data were managed with packages sp [70, 71] and sf [72].

## Results

### Range size and overlap

**Fig. 2:**
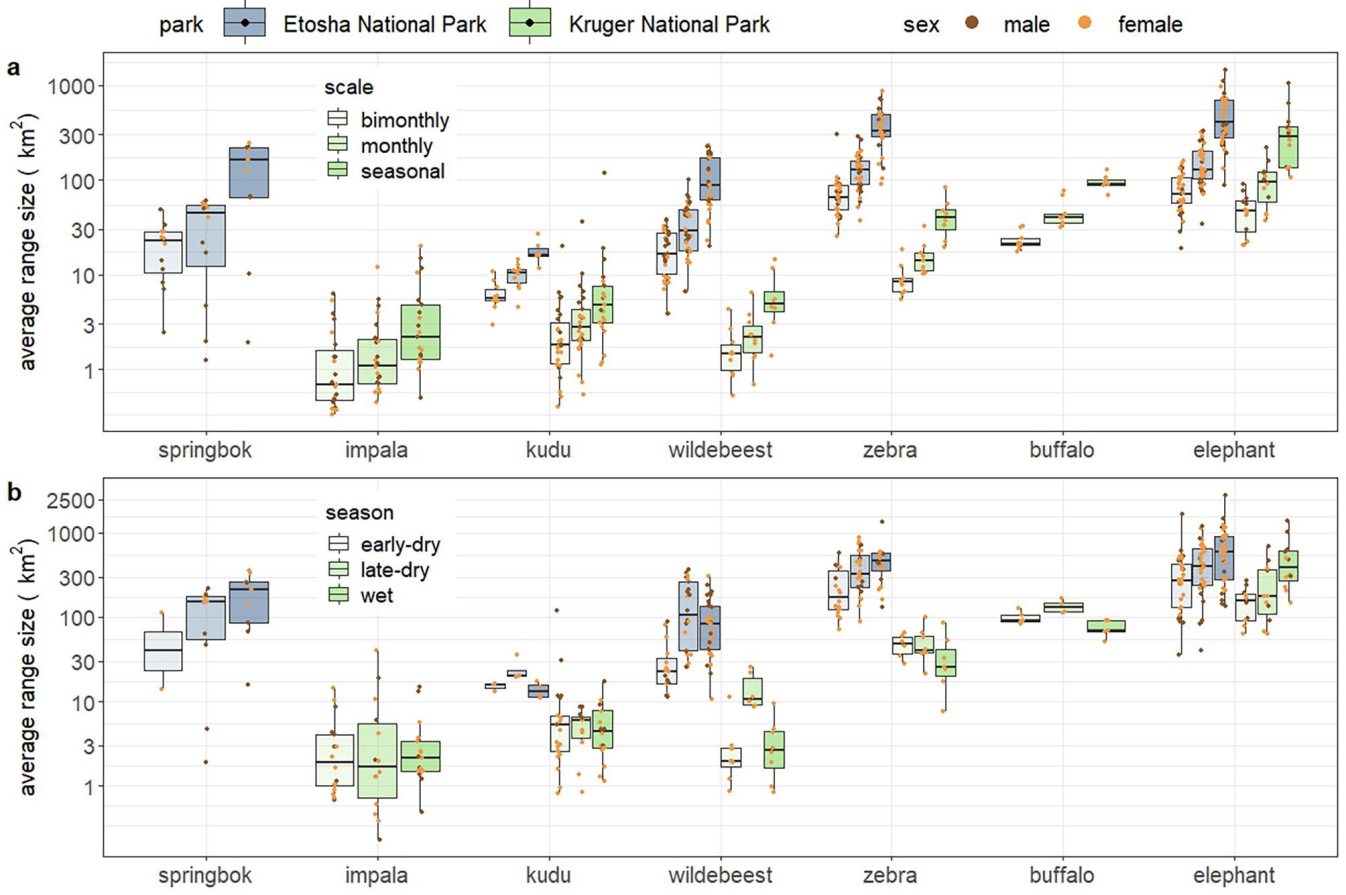
Herbivore range size in Etosha National Park, Namibia and Kruger National Park, South Africa in different temporal scales and seasons, including **a**) bimonthly, monthly and seasonal scales, and **b**) early-dry, late-dry and wet seasons. Range size was calculated with 95% range with *a*-LoCoH (adaptive local convex hull [51]). One data point at bimonthly scale and one at monthly scale from the same female kudu in Kruger were removed from the figure due to very small values (< 0.1 km^2^) for better visualization. Y-axes are log-transformed to better show the differences, and species are ordered along the x-axis based on increasing body mass. Sex of individuals is color-coded

Herbivore range sizes varied with species, parks, temporal scales, seasons, and possibly sexes (Fig. 2; Additional file 2: Table S1 and S2). For species occurring in both parks, range sizes were larger in Etosha than in Kruger at any temporal scale or season (Fig. 2), with elephants having the largest ranges among species. In Etosha, kudu had smallest range sizes among species, and in Kruger, impala, kudu and wildebeest had smaller ranges than other species (Fig. 2). Species with larger range sizes also generally had greater travel distances, shown with NSD (Additional file 1: Supplementary Methods; Additional file 3: Figure S4) and mean daily displacement (Additional file 2: Table S3). For any species by park, range size became larger when the temporal scales were larger, but for some species in Kruger, the differences in range size among time scales were less obvious (e.g., impala and kudu; Fig. 2a). Seasonal differences in range size also varied with species or park (Fig. 2b). Species range sizes in anthrax seasons were not consistently smaller or larger than in other seasons (Fig. 2b). For example, springbok, kudu and buffalo used larger ranges in their anthrax seasons, while wildebeest and elephant had smaller range sizes in their anthrax seasons (Table 1; Fig. 2b). Though not every species had data for both male and female individuals, sex modulated range size for some species. For wildebeest in Etosha and kudu in Kruger, male individuals generally used larger ranges than females (Fig. 2). Male elephants used larger ranges than females in Kruger, while range sizes of male elephants in Etosha had larger variation with some individuals using relatively small areas (Fig. 2). Herbivore ranges in Kruger were located in areas with higher NDVI than in Etosha (Additional file 1: Supplementary Methods; Additional file 3: Figure S5), because Kruger had higher NDVI values than Etosha (mean NDVI estimates in 2010–2020 from each park: 0.424 in Kruger versus 0.281 in Etosha, excluding its salt pans). Range size was negatively associated with NDVI for browsing and grazing herbivores (but not mixed-feeding herbivores; Additional file 1: Supplementary Methods; Additional file 2: Table S4; Additional file3: Figure S6, S7 and S8). Larger body size also correlated with larger range size (except for springbok; Additional file 1: Supplementary Methods; Additional file 3: Figure S7).

**Fig. 3:**
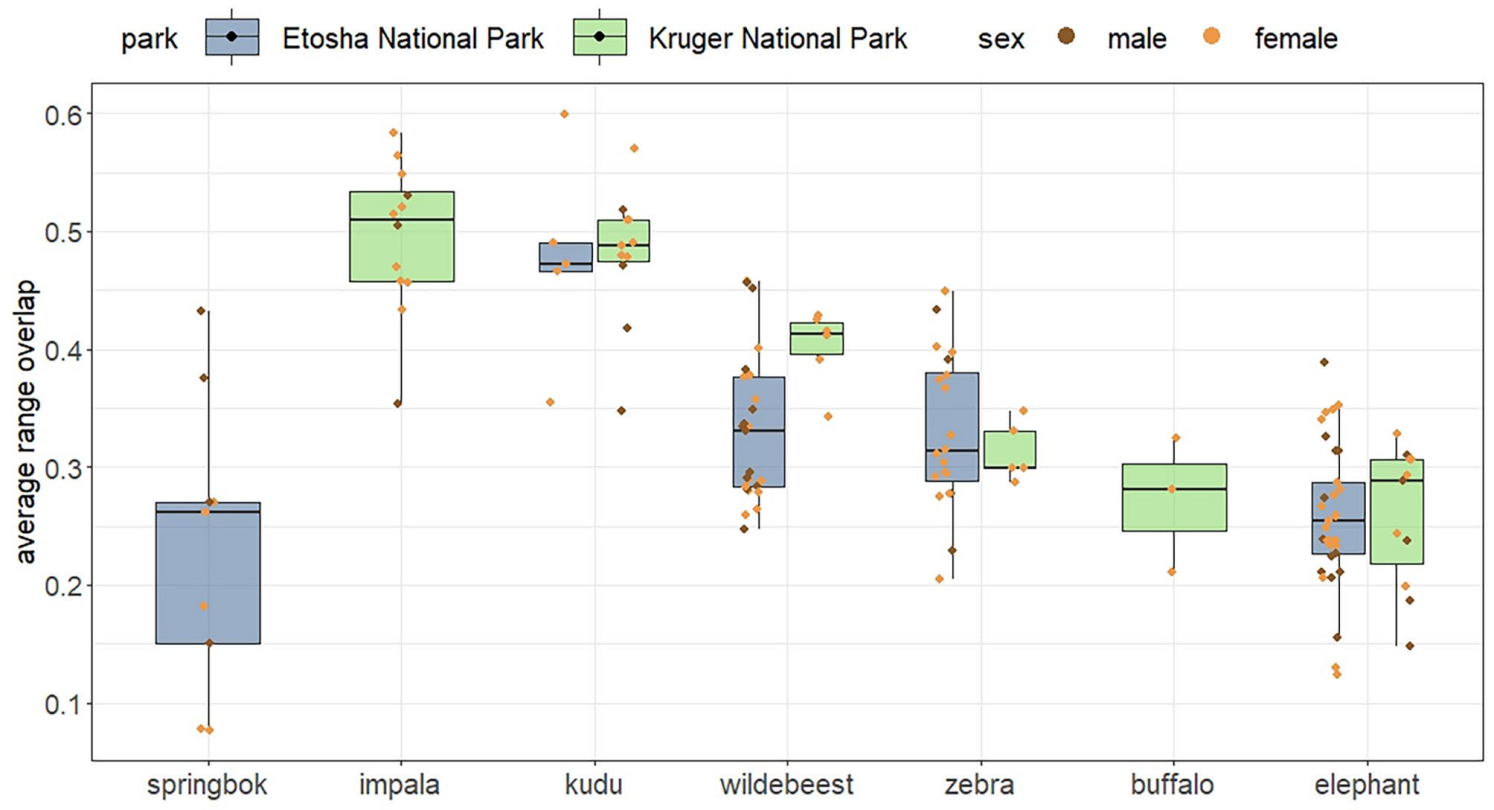
Average proportion of overlap of 95% range from one month to the next for individual herbivores in Etosha National Park, Namibia and Kruger National Park, South Africa. Species are ordered along the x-axis based on increasing body mass, and sex of individuals is color-coded

Individuals of different species differed in their range overlap—in their repeated use of the same areas. However, there were no consistent patterns in overlap for species occurring in both parks, such that no park consistently had more overlap than the other (Fig. 3). Impala and kudu had higher range overlap than other species (Fig. 3), with median overlap proportions close to 0.5, indicating that they repeatedly utilized the same parts of their ranges from one month to the next. Range overlap also varied with seasons, but no consistent patterns were observed comparing the species or parks, or with anthrax seasonality (Additional file 3: Figure S9).

**Fig. 4:**
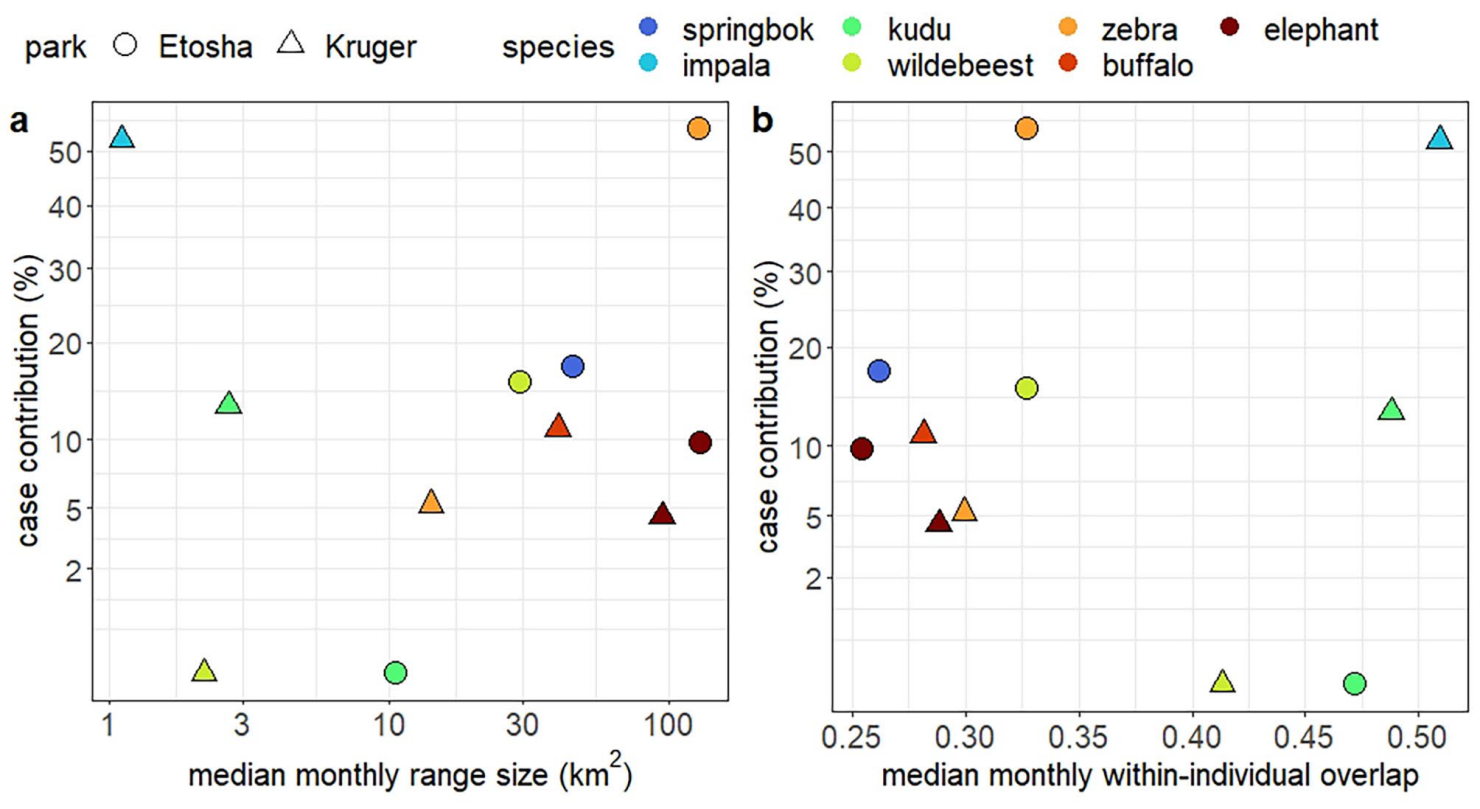
The scatterplots with anthrax outbreak patterns and host space use, including **a**) species contributions to anthrax cases against median monthly range size and **b**) species contributions to anthrax cases against monthly within-individual range overlap. Case contributions were retrieved from central Etosha National Park, Namibia 1976–2014 and northern Kruger National Park, South Africa 2010–2015 (Table 1). Because anthrax cases were barely found for kudu in Etosha and wildebeest in Kruger, their case contributions were set to zero in the calculations. X-axes of plot **a** is log transformed; and y-axes of plot **a** and **b** are square root transformed

Comparing between herbivore ranging behavior and anthrax cases, no significant effect of range size or overlap on species contributions to anthrax cases was detected in either park (Fig. 4; Additional file 2: Table S5), though the sample sizes were small.

### Spatial extent of anthrax mortalities

**Fig. 5:**
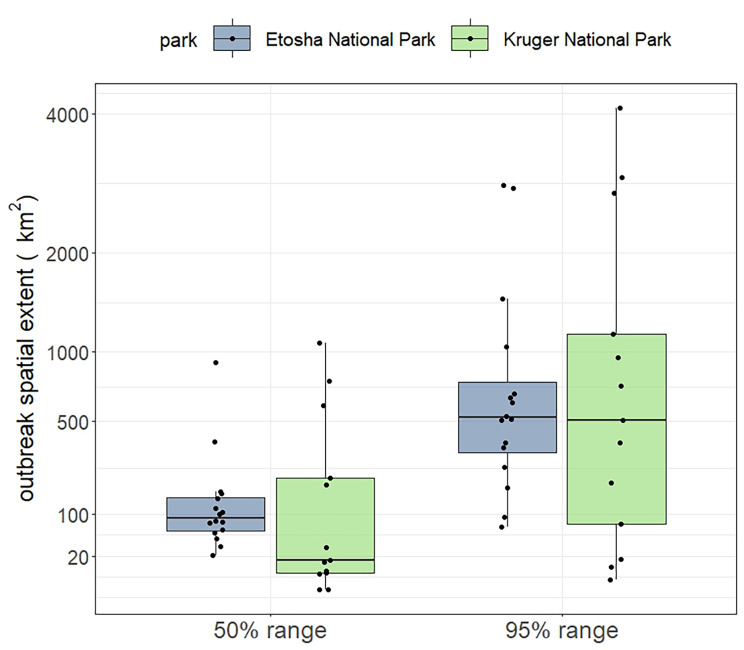
Spatial extent of annual anthrax mortalities in Etosha National Park, Namibia and Kruger National Park, South Africa, including 50% and 95% ranges, calculated with *a*-LoCoH (adaptive local convex hull). Each point is one year from Etosha 1996–2014 and Kruger 1990–2015, with years with fewer than 10 cases removed. The y-axis is square root transformed

The spatial extent of anthrax mortalities was similar between the two parks, although the spatial extent in Kruger was more variable than in Etosha (Fig. 5). The median extent of the 50% range in Etosha was larger than in Kruger, and extent medians of the 95% range were similar between the parks (Fig. 5).

**Fig. 6:**
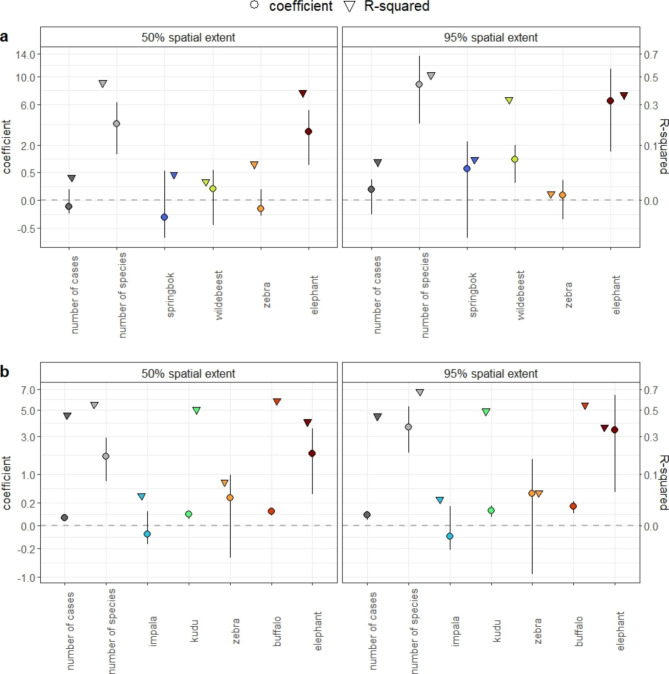
Correlations between spatial extent of annual anthrax mortality (50% and 95% ranges) and tested variables, including outbreak size, number of species in the outbreak, and number of cases for common host species (Table 1), for **a**) Etosha National Park, Namibia and **b**) Kruger National Park, South Africa. The coefficients and R-squared values were calculated by linear regressions, with one variable in a regression. The circles are means of the coefficients; the ranges are 95% confidence intervals

The results of linear regressions using the 50% and 95% spatial extent were very similar (Fig. 6), with wildebeest in Etosha as the only obvious difference. In Etosha we detected significant relationships in the spatial extent of anthrax mortalities and the number of wildebeest (but not for the 50% spatial extent) and elephant cases, and number of species contributing to the outbreak (Fig. 6a; Additional file 2: Table S6). The spatial extent of outbreaks in Etosha was not related to total number of cases detected or number of cases of other common host species (Fig. 6a; Additional file 2: Table S6). In Kruger, when anthrax outbreaks occurred over a large spatial extent, there were also high numbers of cases and species involved; spatial extent was positively linked with case numbers of kudu, buffalo and elephant, but not with case numbers of impala or zebra (Fig. 6b; Additional file 2: Table S6). For those predictors showing significant relationships, their R-squared values were higher than 0.35 (Fig. 6; Additional file 2: Table S6).

## Discussion

This study provides insights on differences in range sizes for multiple herbivore species in two savanna ecosystems with different anthrax outbreak patterns in southern Africa. Our goal was to assess if host space use could be linked to anthrax dynamics at two different scales: (1) if the main host species were those with smaller range sizes and more range overlap, and (2) if outbreak spatial extent was associated with anthrax cases in highly mobile species. Herbivore range sizes differed with species and parks, with individuals generally using larger ranges in Etosha than in Kruger. Though the variation in range may be related to anthrax outbreak dynamics, there was no consistent pattern linking range size to anthrax mortality risk across the two study systems, possibly due other factors not considered here. The spatial extent of anthrax outbreaks was positively linked with case numbers of high mobility species with large ranges. These species may play an important role in the spread of outbreaks on the landscape, in particular species that may otherwise contribute relatively few cases to anthrax outbreaks, such as elephant. Thus, while we did not detect a simple relationship between range size and anthrax risk that applied across our two study systems, average range sizes of particular species may play a role in the spatial extent of outbreaks.

Herbivores in Etosha used larger ranges than in Kruger across any temporal scales or seasons considered, despite the movement data being assembled from different studies which could have spatially or temporally confounding effects. The differences in range size for grazing and browsing herbivores between the parks can be attributed to differences in resource availability; for example, Etosha has lower water availability and lower vegetation productivity than Kruger [35, 38, 39], and thus, herbivores may use larger areas in Etosha to access sufficient nutritional resources.

Outbreak patterns and transmission mechanisms in wildlife-disease systems may vary across locations and scales [35, 73], which makes it challenging to determine general risk patterns across regions. While a larger range may mean a higher probability of encountering a high-risk area when risk is heterogeneously distributed across a landscape, small range size was previously hypothesized to heighten anthrax risk [44]. Our findings indicate that anthrax cases were not linearly associated with range size or range overlap. However, these range size differences as well as differences in an individual’s amount of range overlap over time may have implications for anthrax infection patterns between study areas. Commonly infected host species (impala and kudu) in Kruger used smaller areas and had higher range overlap (Figs. 2 and 3; Additional file 3: Figure S9), implying that when outbreaks occur within their range, they are likely to be exposed or repeatedly exposed to the pathogen, due to revisitations. While range sizes appear to be potentially relevant to exposure in Kruger, in Etosha the same pattern was not observed. In Etosha kudu had the smallest range sizes and highest range overlap but little contribution to anthrax cases, while zebra, the most commonly infected host, utilized relatively large ranges with intermediate range overlap.

Species differences in the contribution to anthrax outbreaks could be driven by differences in host density, behavior, exposure, or susceptibility [4, 34, 35, 40, 74, 75]. While these factors contribute to infection patterns, they cannot wholly explain the observed anthrax patterns, and range size may potentially contribute to some of the variation observed. The lack of consistent patterns may be attributed to a limited influence of range size on anthrax transmission or to other factors that have a larger effect on exposure risk, such as variation in anthrax risk among habitats or differences in host susceptibility. Anthrax risk in Etosha is highest in grassland habitats [4] that are rarely used by browsing hosts such as kudu, so there may be relatively little risk of anthrax exposure for kudu in Etosha, regardless of their range sizes and degree of overlap. Similarly, in Kruger, wildebeest had ranges sizes similar to impala and kudu, but this species is rarely present in the highest anthrax incidence region, whereas wildebeest in Etosha regularly use the high incidence area and contribute steadily to anthrax cases. Thus, understanding the spatial scale of anthrax risk across a heterogeneous landscape is important in assessing risk to species occurring in that landscape. These patterns suggest that whether herbivore species are the main anthrax host species in a location is not simply a function of their range sizes or space use but is modulated by other factors. These include degree of risk in the habitats they select [4, 76], the behaviors conducted at high-risk sites for disease transmission [40] and their innate susceptibility [29]. Nevertheless, our results from Kruger suggest that an evaluation of range size may improve our understanding of infection dynamics.

The spatial extent of anthrax outbreaks was related to case numbers of some host species (i.e., kudu, wildebeest, buffalo, and elephant) but not others (i.e., springbok, impala, and zebra) in the two parks. The positive correlations in outbreak spatial extent with case numbers in certain species could be because wider outbreaks occur when host species with high mobility (e.g., buffalo and elephant) are involved, especially in Kruger, where the ranges for some species are restricted by perennial rivers. Though elephant, as a secondary host species, has a limited contribution to anthrax mortalities in both parks (< 10% of cases; Table 1), their large range sizes as well as long-distance movement may facilitate outbreak spread over larger areas if they live a few days after exposure, or if they release more spores into the environment due to their larger body mass than small-bodied species such as springbok or impala. This pattern may also explain why we observe more complex correlative relationships in the timing of cases between elephant and other species in Kruger [35]. Notably, species showing positive correlations with outbreak spatial extent tend to die of anthrax in dry seasons [35], suggesting the dry season outbreaks may also be affected by changes in host susceptibility [77]. Another possible explanation for the positive correlations between spatial extent and case numbers in particular species is that anthrax mortality distributions differ with host species (Additional file 3: Figure S1). For example, kudu and buffalo cases in Kruger and elephant cases in both parks do not always occur in the highest incidence areas (central Etosha and northernmost Kruger), and as a result, larger spatial extent can be observed when these species are involved in an outbreak (Additional file 3: Figure S2). This pattern is more evident in Kruger, where more species and cases were affected when outbreaks covered larger areas.

Transmission of environmentally transmitted pathogens can be affected by variation in the host, the pathogen, and the environment [78]. When a pathogen can infect a wide range of host species, this adds even more complexity to understanding patterns and processes underlying outbreaks. Previous work has shown the importance of host behavior, density, exposure frequency, and immune response in affecting these outbreak patterns [4, 35, 40, 46, 79]. Results of our study suggest that patterns in animal space use vary with species and park, attributed to species feeding habits and body sizes, and differences in resource availability between the parks. Though not every species following the same trend linking space use and anthrax outbreaks, variation in herbivore space use may contribute to the disease dynamics, with small range sizes potentially leading to higher anthrax risk in Kruger and larger range sizes contributing to larger outbreak extent in both parks. The importance of space use alone, independent of other sources of variation among hosts in their ecology, physiology, immunity, or behavior could be disentangled with additional study. Our results suggest that linking host movements and disease dynamics may be a fruitful avenue for future research, with implications beyond anthrax, warranting future empirical and theoretical work to isolate the effects of host range size on disease dynamics.

## Conclusions

Our study shows that herbivore range size varies among species and within species, and that this variation in range size may have implications for disease dynamics. Species with different range sizes and range overlap may experience variation in anthrax exposure risk, dependent on spatial patterns in how risk is distributed across a landscape. This variation suggests that the scale of exposure risk is important to consider in assessing disease risk to a species, and the presence of disease in an area does not necessarily mean it is homogenously distributed across that area. How pathogen reservoirs are distributed across a landscape—and how hosts interact with those reservoirs when moving across those landscapes—is an important aspect of risk assessment for wildlife diseases. We do find evidence that secondary host species with large ranges and high mobility may facilitate the spread of an outbreak from a localized area out across a landscape. While additional research could help isolate movement-specific aspects of disease risk, our study shows that host range sizes and range overlaps have the potential to influence disease outbreak dynamics.

## Electronic supplementary material

Below is the link to the electronic supplementary material.

Supplementary Material 1

Supplementary Material 2

Supplementary Material 3

## Data Availability

Coordinates of anthrax mortalities and elephant movement data in this study are not publicly available due to potential sensitivity. Movement data on springbok and zebra (9 individuals) in Etosha are available from Movebank (https://www.movebank.org/), and data on wildebeest, zebra and buffalo in Kruger are available from AfriMove (https://afrimove.org/) Thinned movement data (excluding elephant datasets) and analysis code are available from the Dryad Digital Repository (10.5061/dryad.rn8pk0pf4).

## Abbreviations

*a*-LoCoH: Adaptive local convex hull
GLMM: Generalized linear mixed model
GPS: Global positioning system
MODIS: Moderate Resolution Imaging Spectroradiometer
NASA: National Aeronautics and Space Administration
NDVI: Normalized Difference Vegetation Index
