# Supplementary material for: Variation in herbivore space use: comparing two savanna ecosystems with different anthrax outbreak patterns in southern Africa
Source: Mov Ecol. 2023 Jul 31;11:46. doi: 10.1186/s40462-023-00385-2 (PMC10392021; doi:10.1186/s40462-023-00385-2)
Supplement: Supplementary file 1 — Supplementary Material 1 [file 40462_2023_385_MOESM1_ESM.pdf]

Supplementary Methods

Any use of trade, firm, or product names is for descriptive purposes only and does not imply endorsement by the U.S. Government.

Net squared displacement

We calculated net squared displacement (NSD) time-series to evaluate whether large space use was linked with long travel distance (i.e., our NSD measure), using data of individuals with more than three-season data for springbok (*Antidorcas marsupialis*), greater kudu (*Tragelaphus strepsiceros*), blue wildebeest (*Connochaetes taurinus*), plains zebra (*Equus quagga*) and African elephant (*Loxodonta africana*) in Etosha National Park, Namibia, and impala (*Aepyceros melampus*), kudu, wildebeest, zebra, African buffalo (*Syncerus caffer*) and elephant in Kruger National Park, South Africa. Preparation of the movement dataset can be seen in the Methods section. The calculation of NSD time-series was squared distances based on the comparisons between the relocations and the first data point of each individual. NSD was calculated using R package amt (Signer et al. 2019).

Based on NSD time-series and maximal NSD values, for species tracked in both parks, individuals in Etosha generally had larger NSD than in Kruger, which was congruent with the patterns in range sizes (Additional file 3: Figure S4). Springbok in Etosha also had large NSD, while impala in Kruger had small NSD, and magnitude of NSD of buffalo was between the two species (Additional file 3: Figure S4).

Species differences in range size and resource availability

We evaluated whether range sizes varied with resource availability using a remotely sensed index of vegetation greenness and biomass, Normalized Difference Vegetation Index (NDVI), to assess resource availability. This index is used widely for spatiotemporal dynamics of photosynthetically absorbed radiation and allows an estimation of greenness or the amount of chlorophyll in vegetation cover (Tucker et al. 1985, Du Plessis 1999). NDVI is broadly useful at showing resource productivity, despite not reflecting the range of forage availability such as the herbaceous layer beneath tree cover reflected in woodlands, and brown foliage herbivores may consume (Treydte et al. 2013). We extracted NDVI from Moderate Resolution Imaging Spectroradiometer (Terra MODIS; MOD13Q1) with spatial and temporal resolution as 250 × 250 m and 16 days starting at the first day of each year (Didan 2015). We extracted NDVI values in seasonal 95% ranges for each individual, and calculated an average NDVI value for each individual by season.

We tested whether range size variation between the two parks could be described by species identity and resource availability by fitting seasonal range area to a log-linked gamma generalized linear mixed model (GLMM), including individuals with at least three seasons of data. The fixed effect predictors in the model included species and the interaction between species and Normalized Difference Vegetation Index (NDVI; a remote-sensing index of

vegetation greenness or biomass), with individual and season as random variables ($N = 109$ individuals and 556 individual-seasons). The random variable season had three categories (wet, early-dry and late-dry). Here we included season as a random effect and not as a fixed effect for several reasons: our goal was not to detect seasonal differences, species data were not collected in the same years adding interannual noise to a seasonal comparison, and seasonal variation is captured within NDVI, a variable we were interested in as a fixed effect. The GLMM is shown with the following equations:

$$\text{Area}_{ijk} = \text{Gamma}(\mu_{isk}, \tau)\,, \quad (1)$$

$$\log(\mu_{ijk}) = \beta_0 + \beta_1 \times \text{species}_{isk} + \beta_2 \times \text{species}_{isk} \times \text{NDVI}_{isk} + \text{individual}_k + \text{season}_k + \varepsilon_k\,, \quad (2)$$

where Gamma is the gamma distribution with mean μ_{isk} and dispersion parameter τ. The coefficient β_0 is the intercept, β_1 is the coefficient for the fixed effect of species, and β_2 is the coefficient for the interaction between species and NDVI (different species could have different coefficients for NDVI). In addition, individual_k and season_k are random intercepts for different individuals and seasons, respectively, with residual ε_k. Herbivore range sizes and coefficients of the GLMM were compared with body size and feeding strategies.

We evaluated whether range size or effect of NDVI on range size varied with body mass or feeding habits of the species. We retrieved NDVI effects (coefficients) on range size from the generalized linear mixed model, and predicted range size for the seven species using the medians

of NDVI values from Etosha and Kruger and compared the effects and predicted range with body mass and diet selection (percentage of C4 in diet). Data on body mass and feeding habits were retrieved from literature (Cumming and Cumming 2003, Sponheimer et al. 2003, Codron et al. 2006, Codron et al. 2007; Additional file 2: Table S6). We performed the gamma GLMM using package glmmTMB (Brooks et al. 2017), estimated predictions using package ggeffects (Lüdecke 2018), and tested variations in residuals and ensured that assumptions of the GLMM were not violated using simulated residuals generated by package DHARMa (Hartig 2021).

Herbivore ranges were located in areas with higher NDVI in Kruger than in Etosha (Additional file 3: Figure S5). In Etosha, herbivores selecting woodland habitats (i.e., kudu and elephant) used ranges with higher NDVI than the other species (Additional file 3: Figure S5). In Kruger, a relationship between NDVI and habitat preference among species was not apparent, but elephant and buffalo utilized ranges with higher NDVI than the other species (Additional file 3: Figure S5).

Range size was negatively associated with NDVI for kudu, wildebeest, zebra and buffalo, with animals using greener habitats having smaller ranges, but no significant correlation was detected for springbok, impala or elephant (gamma GLMM; Additional file 2: Table S4; Additional file 3: Figure S6). The largest effect of NDVI on space use was observed for wildebeest, with a strong negative effect of NDVI on range size (Additional file 3: Figure S6).

The residuals of the GLMM did not show heterogeneity in variation between the two parks (Levene's test: $p > 0.05$; Additional file 3: Figure S10), indicating that habitat differences captured by NDVI may in part explain differences in range size between the two ecosystems.

We found that body size was positively correlated with the GLMM predicted range size, using medians of NDVI estimates from herbivore ranges in Etosha (0.259) and Kruger (0.439) (Additional file 3: Figure S7a). Springbok was an exception which had large ranges while having the smallest body size (Additional file 3: Figure S7a). Excluding springbok, range size and body mass were correlated (both variables were log-transformed; $N = 6$ species; NDVI from Etosha: 95% CI of the linear regression slope: 0.25-2.45, R^2: 0.68; NDVI from Kruger: 95% CI of the slope: 0.49-2.56, R^2: 0.76). However, the effects of NDVI on range size was not modulated by body mass (Additional file 3: Figure S8a). While no pattern was detected between range sizes and C4 percentages in diets (Additional file 3: Figure S7b), feeding habits alter the relationship between NDVI and range size (Additional file 3: Figure S8b). Species which tend to graze or browse had negative effects of NDVI on range sizes, while there was no significant effect for mixed-feeders (Additional file 3: Figure S8b)

Supplementary References

Brooks, M. E., K. Kristensen, K. J. Van Benthem, A. Magnusson, C. W. Berg, A. Nielsen, H. J. Skaug, M. Machler, and B. M. Bolker. 2017. glmmTMB balances speed and flexibility

among packages for zero-inflated generalized linear mixed modeling. The R journal **9**:378-400.

Codron, D., J. Codron, J. A. Lee-Thorp, M. Sponheimer, D. De Ruiter, J. Sealy, R. Grant, and N. Fourie. 2007. Diets of savanna ungulates from stable carbon isotope composition of faeces. Journal of Zoology 273:21-29.

Codron, J., J. A. Lee-Thorp, M. Sponheimer, D. Codron, R. C. Grant, and D. J. de Ruiter. 2006. Elephant (Loxodonta africana) Diets in Kruger National Park, South Africa: Spatial and Landscape Differences. Journal of Mammalogy 87:27-34.

Cumming, D. H., and G. S. Cumming. 2003. Ungulate community structure and ecological processes: body size, hoof area and trampling in African savannas. Oecologia 134:560-568.

Didan, K. 2015. MOD13Q1 MODIS/Terra vegetation indices 16-day L3 global 250m SIN grid V006. NASA EOSDIS Land Processes DAAC **10**:415.

Du Plessis, W. 1999. Linear regression relationships between NDVI, vegetation and rainfall in Etosha National Park, Namibia. Journal of Arid Environments **42**:235-260.

Hartig, F. 2021. DHARMa: residual diagnostics for hierarchical (multi-level/mixed) regression models. R package version 0.4.4.

Lüdecke, D. 2018. ggeffects: Tidy data frames of marginal effects from regression models. Journal of Open Source Software **3**:772.

Signer, J., J. Fieberg, and T. Avgar. 2019. Animal movement tools (amt): R package for managing tracking data and conducting habitat selection analyses. Ecology and Evolution 9:880-890.

Sponheimer, M., J. A. Lee-Thorp, D. J. DeRuiter, J. M. Smith, N. J. van der Merwe, K. Reed, C. C. Grant, L. K. Ayliffe, T. F. Robinson, C. Heidelberger, and W. Marcus. 2003. Diets of Southern African Bovidae: Stable Isotope Evidence. Journal of Mammalogy 84:471-479.

Treydte, A. C., S. Baumgartner, I. M. A. Heitkönig, C. C. Grant, and W. M. Getz. 2013. Herbaceous Forage and Selection Patterns by Ungulates across Varying Herbivore Assemblages in a South African Savanna. PloS One **8**:e82831.

Tucker, C. J., C. L. Vanpraet, M. Sharman, and G. Van Ittersum. 1985. Satellite remote sensing of total herbaceous biomass production in the Senegalese Sahel: 1980–1984. Remote Sensing of Environment **17**:233-249.
