# Supplementary material for: Variation in herbivore space use: comparing two savanna ecosystems with different anthrax outbreak patterns in southern Africa
Source: Mov Ecol. 2023 Jul 31;11:46. doi: 10.1186/s40462-023-00385-2 (PMC10392021; doi:10.1186/s40462-023-00385-2)
Supplement: Supplementary file 2 — Supplementary Material 2 [file 40462_2023_385_MOESM2_ESM.pdf]

Supplementary Tables

Table S1. Descriptive statistics of herbivore range sizes at three temporal scales in Etosha National Park, Namibia and Kruger National Park, South Africa for potential anthrax host species, including springbok (*Antidorcas marsupialis*), impala (*Aepyceros melampus*), greater kudu (*Tragelaphus strepsiceros*), blue wildebeest (*Connochaetes taurinus*), plains zebra (*Equus quagga*), African buffalo (*Syncerus caffer*) and African elephant (*Loxodonta africana*). The units for range sizes are km^2.

species	number of individuals	mean	median	minimum	maximum	standard deviation	interquartile range
bimonthly interval in Etosha National Park							
springbok	12	21.0	22.7	2.5	48.1	13.0	17.6
kudu	10	6.3	5.7	3.0	10.9	2.2	1.8
wildebeest	34	19.2	16.6	3.8	37.8	9.8	17.0
zebra	37	72.2	64.6	25.1	306.5	44.8	39.7
elephant	34	81.8	71.8	19.1	161.0	37.7	49.3
bimonthly interval in Kruger National Park							
impala	23	1.5	0.7	0.3	6.3	1.7	1.1
kudu	27	2.9	1.8	0.0	19.9	3.8	1.9
wildebeest	10	1.7	1.5	0.5	4.3	1.1	0.8
zebra	9	9.2	8.3	5.5	18.5	4.0	2.5
buffalo	9	23.3	21.3	17.3	32.8	5.4	3.9
elephant	12	48.1	47.6	20.4	90.2	22.5	32.0
monthly interval in Etosha National Park							
springbok	12	34.5	45.2	1.2	60.4	23.5	40.6
kudu	10	9.9	10.5	4.5	14.4	2.9	3.1
wildebeest	34	35.6	29.3	6.5	101.2	20.9	30.2
zebra	37	131.1	128.3	36.8	284.1	54.2	68.7
elephant	34	159.7	129.3	33.9	325.3	79.2	99.4

monthly interval in Kruger National Park							
impala	22	2.1	1.1	0.4	12.0	2.6	1.4
kudu	26	4.5	2.7	0.0	36.0	6.8	2.3
wildebeest	10	2.5	2.2	0.7	6.5	1.7	1.4
zebra	9	15.9	14.2	10.2	32.0	6.9	5.7
buffalo	9	46.0	40.3	31.2	76.6	16.1	8.8
elephant	12	99.0	95.2	37.2	218.0	52.6	61.3
seasonal interval in Etosha National Park							
springbok	11	140.0	164.3	1.9	244.6	90.3	151.5
kudu	6	17.7	16.1	11.7	26.7	5.2	3.5
wildebeest	30	109.4	87.2	19.8	232.1	64.8	109.0
zebra	28	375.4	330.8	89.6	874.3	189.3	210.0
elephant	34	507.3	412.9	87.8	1457.2	299.7	410.0
seasonal interval in Kruger National Park							
impala	21	4.5	2.2	0.5	20.0	5.3	3.5
kudu	21	11.2	4.8	1.1	119.4	25.2	4.4
wildebeest	8	6.3	5.0	1.4	14.3	4.4	2.9
zebra	8	42.6	40.3	19.3	83.3	20.4	18.7
buffalo	7	95.7	90.5	69.5	129.0	18.4	13.6
elephant	12	342.0	288.0	107.2	1042.8	268.5	234.5

Table S2. Descriptive statistics of herbivore average seasonal range sizes in Etosha National Park, Namibia and Kruger National Park, South Africa for potential anthrax host species, including springbok (*Antidorcas marsupialis*), impala (*Aepyceros melampus*), greater kudu (*Tragelaphus strepsiceros*), blue wildebeest (*Connochaetes taurinus*), plains zebra (*Equus quagga*), African buffalo (*Syncerus caffer*) and African elephant (*Loxodonta africana*). The units for range sizes are km^2.

species	number of individuals	mean	median	minimum	maximum	standard deviation	interquartile range
Etosha National Park							
early-dry (dry) season							
springbok	2	63.2	63.2	14.0	112.3	69.5	49.1
kudu	3	15.1	15.9	13.2	16.2	1.6	1.5
wildebeest	15	31.2	22.4	11.1	89.3	24.7	16.3
zebra	18	241.5	174.2	72.3	584.5	163.9	238.0
elephant	32	327.4	268.2	35.8	1653.1	293.4	298.6
late-dry (semi-dry) season							
springbok	11	121.2	150.3	1.9	218.4	77.2	122.4
kudu	4	23.9	20.1	19.6	36.0	8.1	4.2
wildebeest	24	153.9	106.9	25.6	372.0	116.0	223.6
zebra	23	397.8	324.3	89.6	874.3	225.1	324.2
elephant	31	458.0	397.6	40.1	1209.0	292.4	406.1
wet season							
springbok	9	192.5	211.1	15.8	359.3	121.0	174.9
kudu	4	13.7	13.4	10.8	17.4	3.1	4.2
wildebeest	29	98.5	83.3	10.5	301.2	73.2	95.5
zebra	20	468.7	466.6	132.5	1334.1	252.8	214.8
elephant	33	679.3	589.4	134.3	2820.8	530.7	637.7
Kruger National Park							
early-dry season							

impala	16	3.6	1.9	0.7	14.2	4.0	3.0
kudu	21	11.6	5.3	0.8	119.4	25.5	4.1
wildebeest	8	3.1	1.9	0.9	11.4	3.4	1.0
zebra	6	47.4	48.4	28.3	64.7	14.1	20.1
buffalo	5	99.1	90.5	83.5	127.9	18.3	18.7
elephant	10	153.1	158.7	63.8	270.1	70.2	98.0
late-dry season							
impala	14	6.4	1.7	0.2	40.0	11.0	4.8
kudu	11	5.2	5.9	0.8	8.7	2.6	2.9
wildebeest	6	14.4	10.7	8.6	25.5	7.4	10.2
zebra	8	49.9	40.7	21.2	98.4	23.8	22.3
buffalo	4	134.8	130.1	111.6	167.3	26.3	36.0
elephant	11	250.6	178.6	63.7	701.2	200.6	252.1
wet season							
impala	17	3.6	2.1	0.5	15.0	4.1	1.9
kudu	16	6.0	4.4	1.1	17.4	5.2	5.2
wildebeest	8	3.4	2.6	0.8	9.4	2.8	2.7
zebra	7	35.3	25.3	7.5	86.7	26.8	22.6
buffalo	5	73.9	69.5	50.8	91.3	17.1	23.2
elephant	12	509.8	400.9	146.2	1384.5	368.3	336.1

Table S3. Herbivore average daily displacement in the morning, afternoon and night in Etosha National Park, Namibia and Kruger National Park, South Africa for potential anthrax host species, including springbok (*Antidorcas marsupialis*), impala (*Aepyceros melampus*), greater kudu (*Tragelaphus strepsiceros*), blue wildebeest (*Connochaetes taurinus*), plains zebra (*Equus quagga*), African buffalo (*Syncerus caffer*) and African elephant (*Loxodonta africana*). The units for displacement are km.

species	morning displacement	afternoon displacement	night displacement
		Etosha National Park	
springbok	2.47	2.25	2.69
kudu	2.95	2.66	1.80
wildebeest	3.37	3.64	2.63
zebra	5.63	5.98	4.93
elephant	5.20	5.53	5.33
		Kruger National Park	
impala	0.78	0.82	0.65
kudu	1.13	1.17	0.92
wildebeest	1.11	1.12	0.87
zebra	2.30	2.21	1.77
buffalo	2.99	3.00	2.73
elephant	3.88	3.96	4.03

Table S4. Estimated coefficients for fixed effect variables in the gamma generalized linear mixed model, for evaluating the relationships between herbivore range size and vegetation biomass (reflected by NDVI; Normalized Difference Vegetation Index; a remote-sensing index of vegetation greenness or biomass) in Etosha National Park, Namibia and Kruger National Park, South Africa (Additional file 1: Supplementary Methods). The herbivore species included springbok (*Antidorcas marsupialis*), impala (*Aepyceros melampus*), greater kudu (*Tragelaphus strepsiceros*), blue wildebeest (*Connochaetes taurinus*), plains zebra (*Equus quagga*), African buffalo (*Syncerus caffer*) and African elephant (*Loxodonta africana*). The table shows mean coefficients and lower and upper bounds of 95% confidence intervals.

	mean	lower bound	upper bound
intercept (springbok as the base)			
overall	4.41	2.42	6.40
impala	-2.31	-4.66	0.04
kudu	-1.05	-3.19	1.08
wildebeest	1.06	-0.92	3.05
zebra	2.08	0.08	4.07
buffalo	2.00	-0.36	4.36
elephant	1.74	-0.22	3.69
coefficient for NDVI			
springbok	2.49	-5.03	10.01
Impala	-3.00	-6.38	0.39
kudu	-4.29	-6.70	-1.89
wildebeest	-7.68	-9.16	-6.20
zebra	-3.90	-5.55	-2.24
buffalo	-3.67	-6.25	-1.09
elephant	-0.83	-1.90	0.23

Table S5. Estimated slopes of linear regressions for evaluating the relationships between herbivore range size (square root transformed) or overlap and species contributions to anthrax cases (Table 1) in Etosha National Park, Namibia and Kruger National Park, South Africa. Because anthrax cases were barely found for kudu in Etosha and wildebeest in Kruger, their case contributions were set to zero in the calculations. The table shows mean slopes, lower and upper bounds of slope 95% confidence intervals and R-squared values.

park	predictor	mean	lower bound	upper bound	R-squared
Etosha	range size	3.59	-4.55	11.73	0.40
Kruger	range size	-2.27	-9.30	4.77	0.17
Etosha	overlap	-65.03	-484.25	354.18	0.08
Kruger	overlap	115.47	-83.36	314.30	0.39

Table S6. Estimated slopes of linear regressions for evaluating the relationships between anthrax outbreak spatial extent and number of total cases, number of species involved and number of cases in common host species in Etosha National Park, Namibia and Kruger National Park, South Africa. The table shows mean slopes and lower and upper bounds of 95% confidence intervals. The herbivore species included springbok (*Antidorcas marsupialis*), impala (*Aepyceros melampus*), greater kudu (*Tragelaphus strepsiceros*), blue wildebeest (*Connochaetes taurinus*), plains zebra (*Equus quagga*), African buffalo (*Syncerus caffer*) and African elephant (*Loxodonta africana*). The table shows mean coefficients, lower and upper bounds of slope 95% confidence intervals and R-squared values.

	mean	lower bound	upper bound	R-squared
	50% spatial extent			
	Etosha National Park			
number of total cases	-0.02	-0.12	0.07	0.18
number of species	3.78	1.37	6.20	0.45
number of springbok cases	-0.19	-0.93	0.55	0.02
number of wildebeest cases	0.09	-0.40	0.59	0.01
number of zebra cases	-0.04	-0.16	0.07	0.04
number of elephant cases	3.04	0.80	5.28	0.38
	Kruger National Park			
number of total cases	0.02	0.01	0.04	0.46
number of species	1.81	0.73	2.89	0.55
number of impala cases	-0.03	-0.13	0.07	0.03
number of kudu cases	0.05	0.02	0.08	0.50
number of zebra cases	0.29	-0.40	0.97	0.07
number of buffalo cases	0.08	0.03	0.12	0.58
number of elephant cases	1.96	0.37	3.55	0.40

	95% spatial extent			
	Etosha National Park			
number of total cases	0.08	-0.13	0.29	0.05
number of species	8.71	3.81	13.61	0.51
number of springbok cases	0.66	-0.91	2.23	0.05
number of wildebeest cases	1.08	0.20	1.96	0.33
number of zebra cases	0.02	-0.24	0.27	0.00
number of elephant cases	6.42	1.52	11.31	0.36
	Kruger National Park			
number of total cases	0.04	0.01	0.07	0.45
number of species	3.65	1.97	5.34	0.67
number of impala cases	-0.04	-0.23	0.14	0.03
number of kudu cases	0.09	0.03	0.14	0.49
number of zebra cases	0.39	-0.88	1.66	0.04
number of buffalo cases	0.14	0.05	0.22	0.55
number of elephant cases	3.41	0.43	6.40	0.37

Table S7. Estimates of mean adult female body mass and percentages of C4 in diets in Etosha National Park, Namibia and Kruger National Park, South Africa for study species retrieved from literature.

Species	adult female mass (kg)*	C4 in diet (%)#
springbok (*Antidorcas marsupialis*)	39	23
impala (*Aepyceros melampus*)	60	60
greater kudu (*Tragelaphus strepsiceros*)	160	7
blue wildebeest (*Connochaetes taurinus*)	180	90
African buffalo (*Syncerus caffer*)	450	88
plains zebra (*Equus quagga*)	302	92
African elephant (*Loxodonta africana*)	2275	30

*Adult female body mass data were retrieved from Cumming and Cumming (2003).

#C4 percentages in diet were retrieved from Sponheimer et al. (2003) for springbok, Codron et al. (2007) for impala, wildebeest, kudu, zebra and buffalo, and Codron et al. (2006) for elephant. Elephant C4 percentage was calculated using average of dry and wet seasons.
