# Supplementary material for: Variation in herbivore space use: comparing two savanna ecosystems with different anthrax outbreak patterns in southern Africa
Source: Mov Ecol. 2023 Jul 31;11:46. doi: 10.1186/s40462-023-00385-2 (PMC10392021; doi:10.1186/s40462-023-00385-2)

Supplementary Figures

Figure S1. Overall spatial distribution of anthrax mortalities by species from **a**) 1996 – 2014 in Etosha National Park, Namibia and **b**) 1990 – 2015 in Kruger National Park, South Africa. Anthrax mortality data used for this figure included only cases with coordinates.

Figure S2. Spatial distribution of anthrax mortalities by year and species from **a**) 1996 – 2014 in Etosha National Park, Namibia and **b**) 1990 – 2015 in Kruger National Park, South Africa. Anthrax mortality data used for this figure included only cases with coordinates and years with at least 10 cases.

Figure S3. First reading for each tracked individual from the telemetry data analyzed in this study. The study species included **a**) springbok, kudu, wildebeest, zebra and elephant in Etosha National Park, Namibia and **b**) impala, kudu, wildebeest, zebra, buffalo and elephant in Kruger National Park, South Africa. Some tracked individuals in Kruger sometimes went outside the park, but they still remained in the Greater Limpopo Transfrontier Park.

Figure S4. Net squared displacement (NSD) starting at the first location of the data for **a**) kudu in Etosha National Park, Namibia, **b**) kudu in Kruger National Park, South Africa, **c**) wildebeest in Etosha, **d**) wildebeest in Kruger, **e**) zebra in Etosha, **f**) zebra in Kruger, **g**) elephant in Etosha, **h**) elephant in Kruger, **i**) springbok in Etosha, **j**) impala in Kruger and **k**) buffalo in Kruger. NSD shown with y-axes is log-transformed post plus one.

Figure S5. Average NDVI (Normalized Difference Vegetation Index; a remote-sensing index of vegetation greenness or biomass) based on 95% herbivore individual ranges in Etosha National Park, Namibia and Kruger National Park, South Africa. Species are ordered along the x-axis based on increasing body mass, and sex of individuals is color-coded.

Figure S6. Effects of NDVI (Normalized Difference Vegetation Index; a remote-sensing index of vegetation greenness or biomass) on range size by herbivore species, estimated with a gamma generalized linear mixed model (Additional file 1: Supplementary Methods), shown with **a**) coefficients by species and **b**) predicted effects. The circles of plot a are means of the coefficients; the ranges are 95% confidence intervals. The lines of plot **b** are means of the effects; the shaded areas are 95% confidence intervals for prediction. Y-axis of plot **b** is log-transformed.

Figure S7. Relationships between estimated ranges by species, and **a**) herbivore body mass and **b**) feeding habits. Feeding habits are represented with C4 percentage as an index which reflects proportion of grass in a diet. Estimated ranges were predicted with a gamma generalized linear mixed model, using medians of NDVI (Normalized Difference Vegetation Index; a remote-sensing index of vegetation greenness or biomass) values from Etosha National Park, Namibia and Kruger National Park, South Africa (Additional file 1: Supplementary Methods). The circles and triangles are average predicted range size; the ranges are their 95% confidence intervals. The

lines of plot a are best fitting lines between body mass and range size, excluding springbok. Y-axes of both plots and x-axis of plot **a** are log-transformed. Information of C4 percentages in diets and body mass was retrieved from literature and summarized in Additional file 2: Table S5.

Figure S8. Relationships between effects of NDVI (Normalized Difference Vegetation Index; a remote-sensing index of vegetation greenness or biomass) on range sizes by species estimated with a gamma generalized linear mixed model (Additional file 1: Supplementary Methods), and **a**) herbivore body mass and **b**) feeding habits. Feeding habits are represented with C4 percentage as an index which reflects proportion of grass in a diet. The circles are means of the coefficients; the ranges are their 95% confidence intervals. X-axis of plot **a** is log-transformed. Information of C4 percentages in diets and body mass was retrieved from literature and summarized in Additional file 2: Table S5.

Figure S9. Average proportion of overlap of 95% range from one month to the next by season for individual herbivores in Etosha National Park, Namibia and Kruger National Park, South Africa. An individual-season was removed from range overlap estimation if there were fewer than three pairs of consecutive months. Species are ordered along the x-axis based on increasing body mass, and sex of individuals is color-coded.

Figure S10. Simulated residuals generated with R package DHARMa by Etosha and Kruger National Parks from the gamma generalized linear mixed model (Additional file 1:

Supplementary Methods). The values of DHARMa residuals represent the proportion of simulated residuals lower than the residuals from the model.

Figure S1

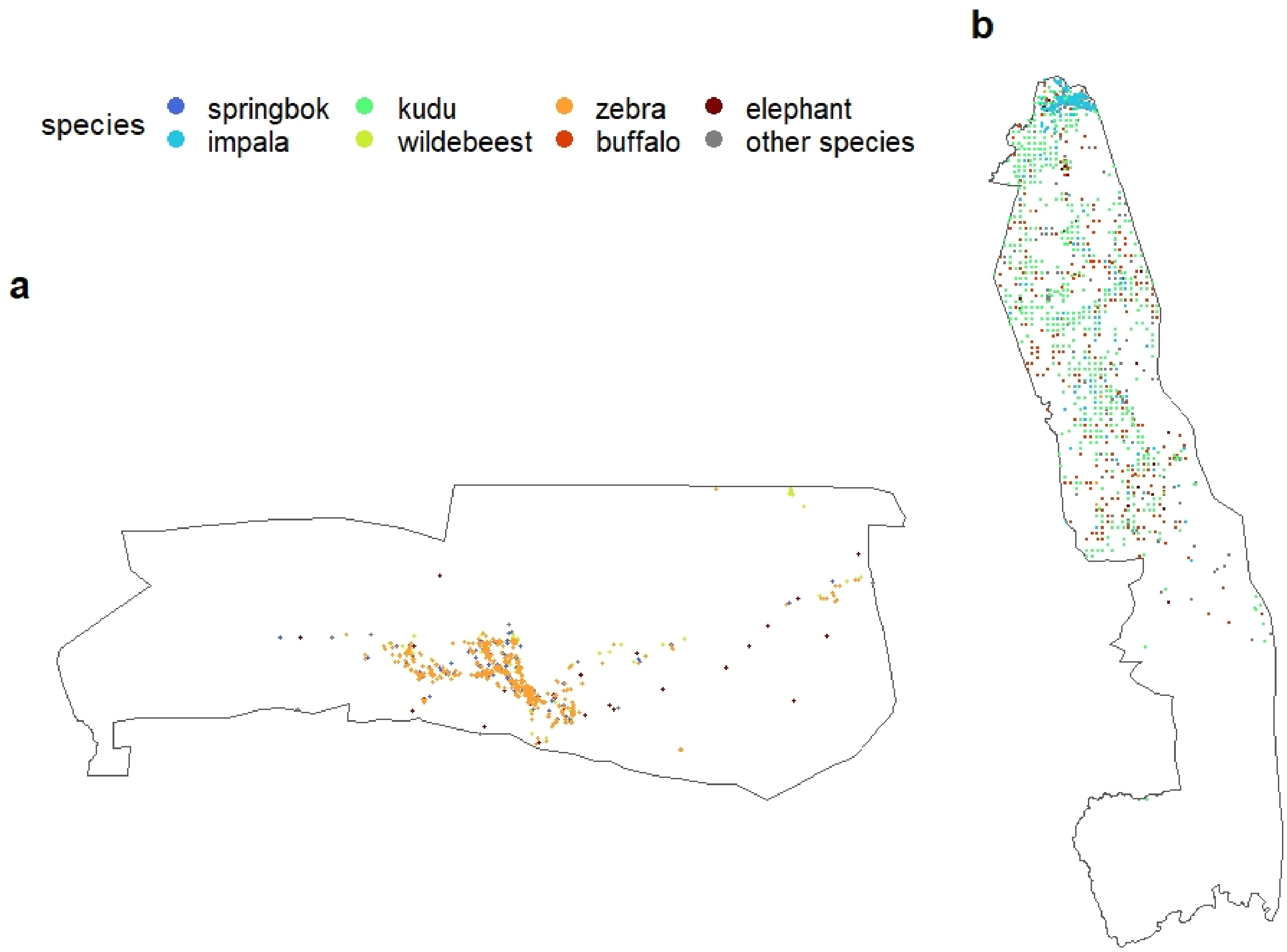

species
springbok
impala
kudu
wildebeest
zebra
buffalo
elephant
other species
a
b

Figure S2

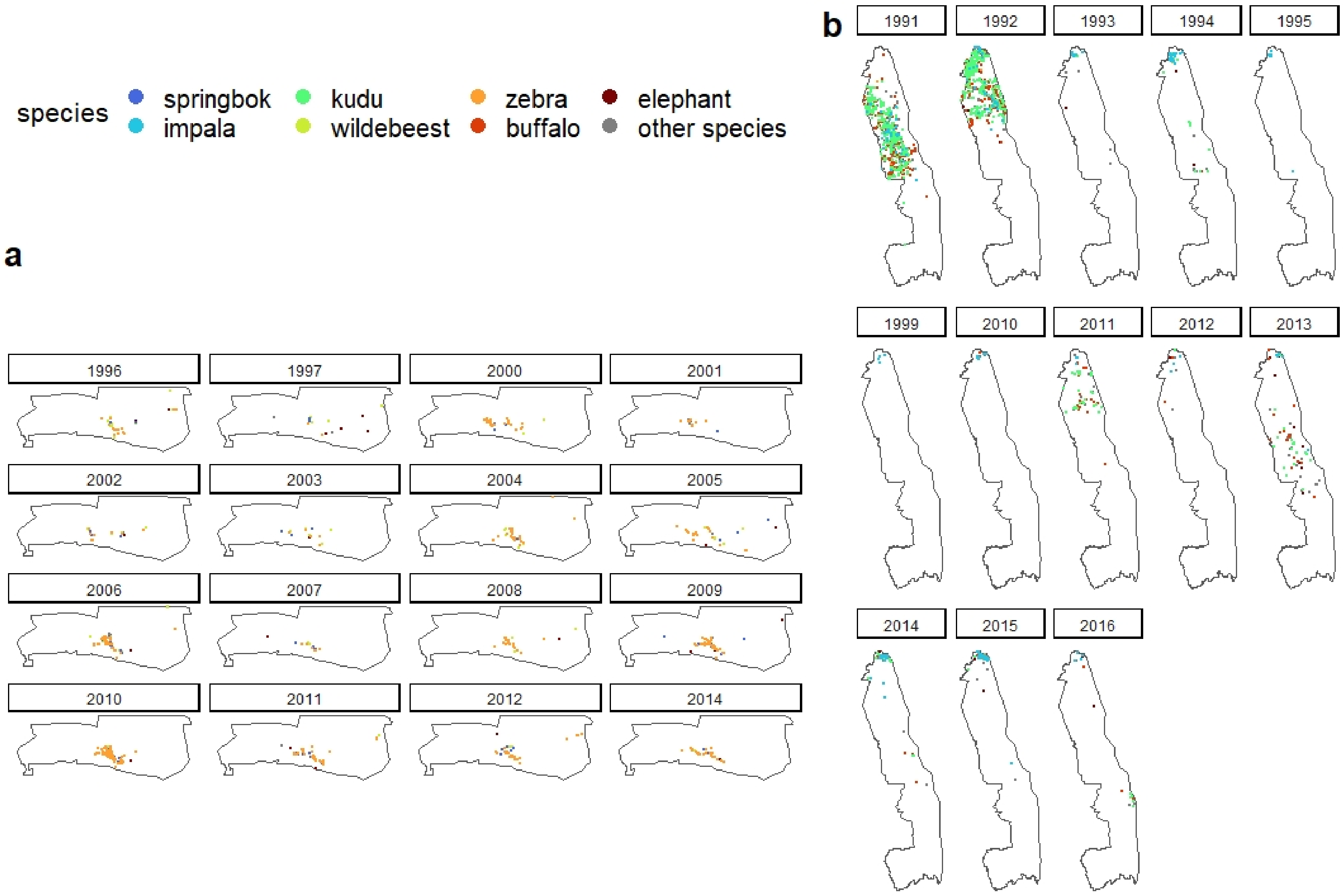
species
springbok
impala
kudu
wildebeest
zebra
buffalo
elephant
other species
a
1996
1997
2000
2001
2002
2003
2004
2005
2006
2007
2008
2009
2010
2011
2012
2014
b
1991
1992
1993
1994
1995
1999
2010
2011
2012
2013
2014
2015
2016

Figure S3

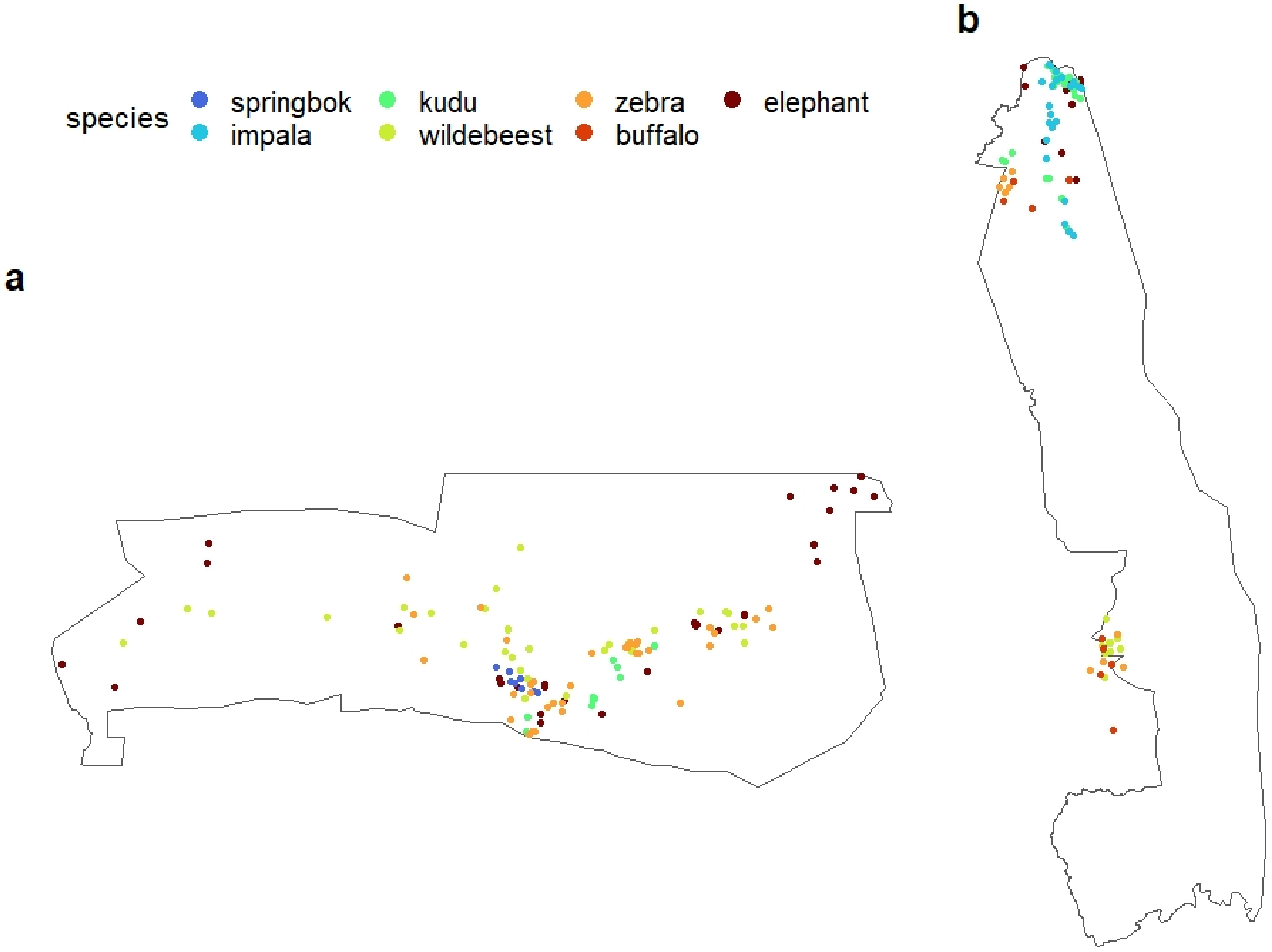
b
species
springbok
impala
kudu
wildebeest
zebra
buffalo
elephant
a

Figure S4

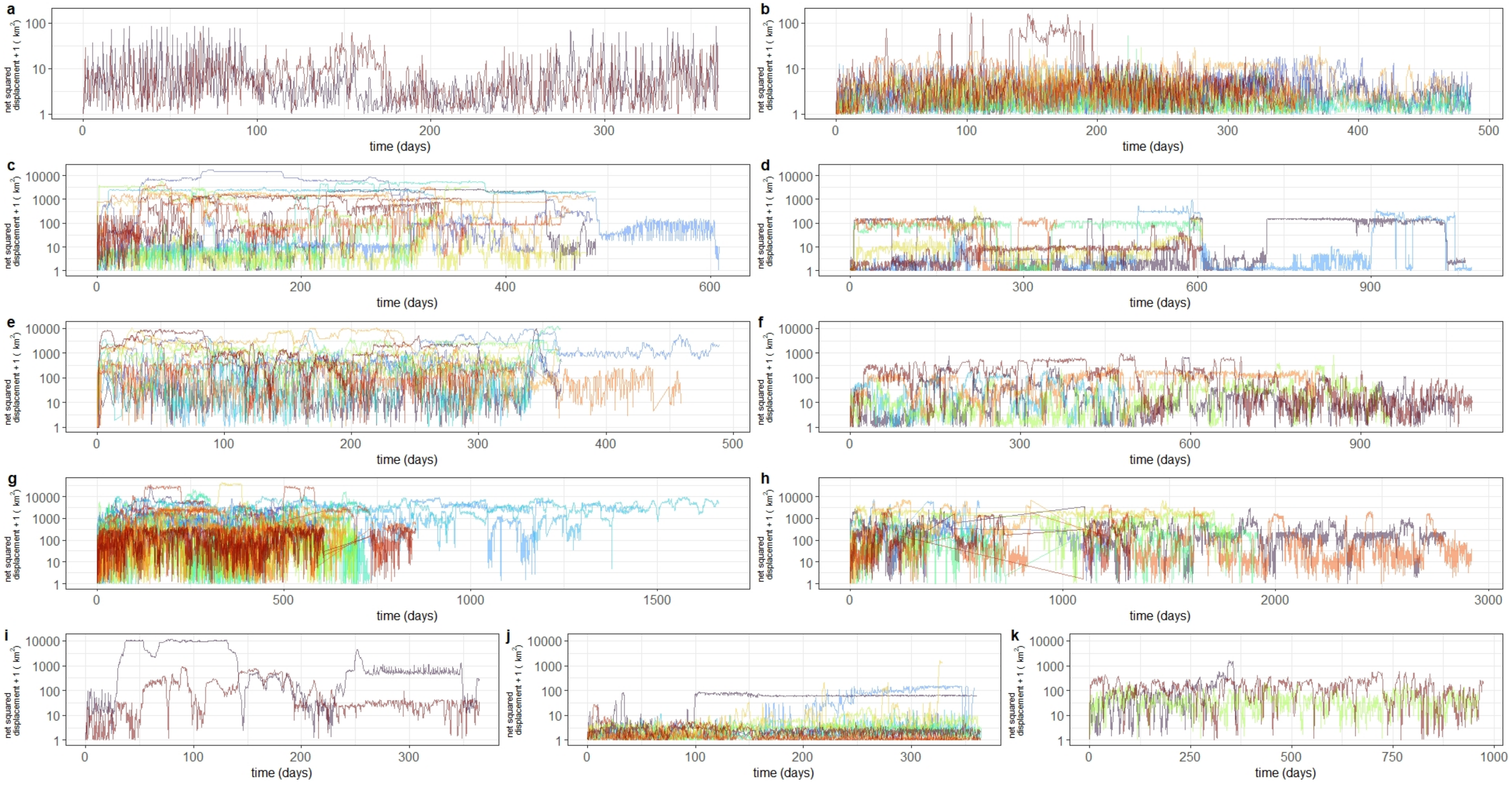

Figure S5

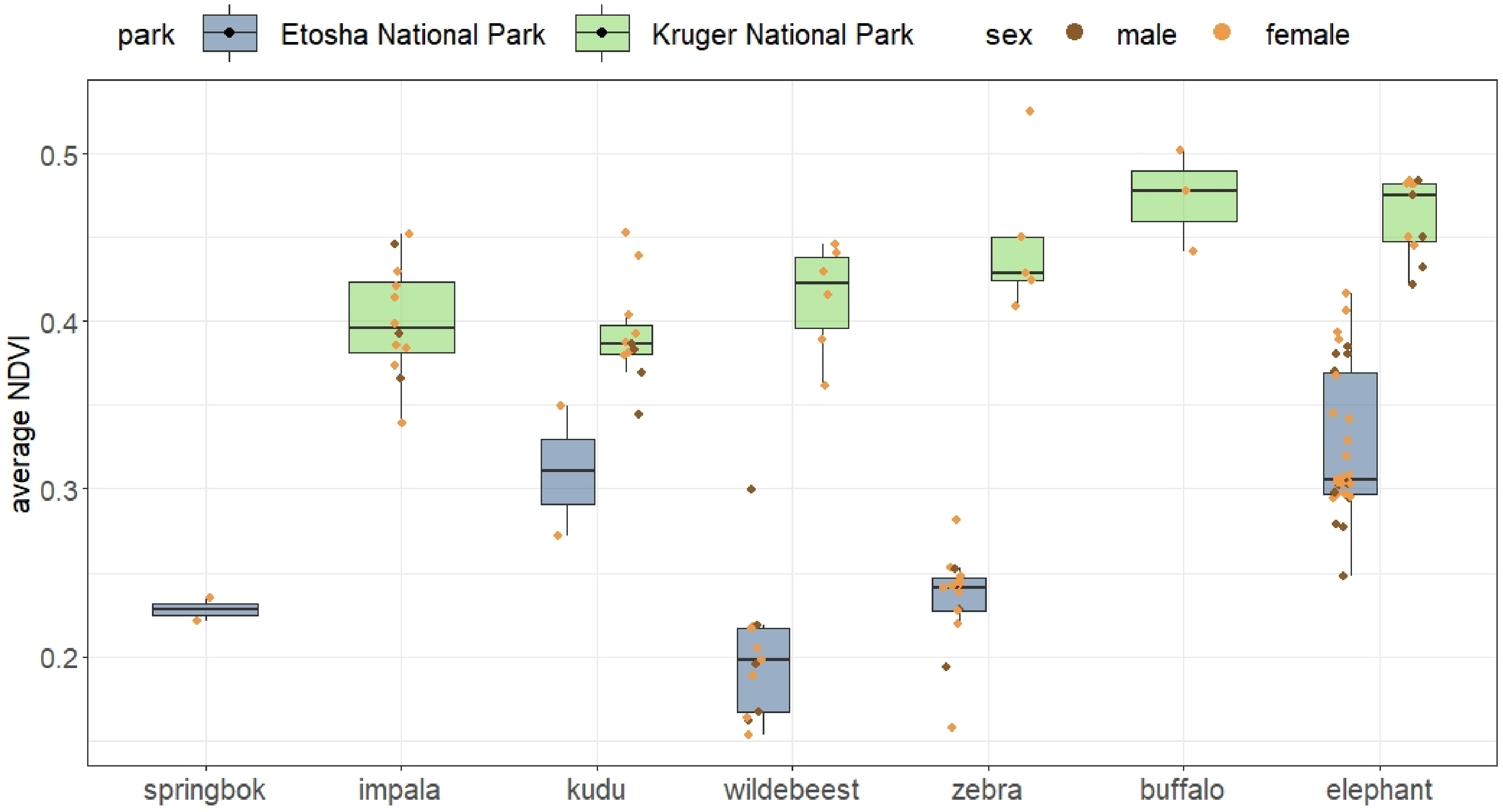
park
Etosha National Park
Kruger National Park
sex
male
female
average NDVI
0.5
0.4
0.3
0.2
springbok
impala
kudu
wildebeest
zebra
buffalo
elephant

Figure S6

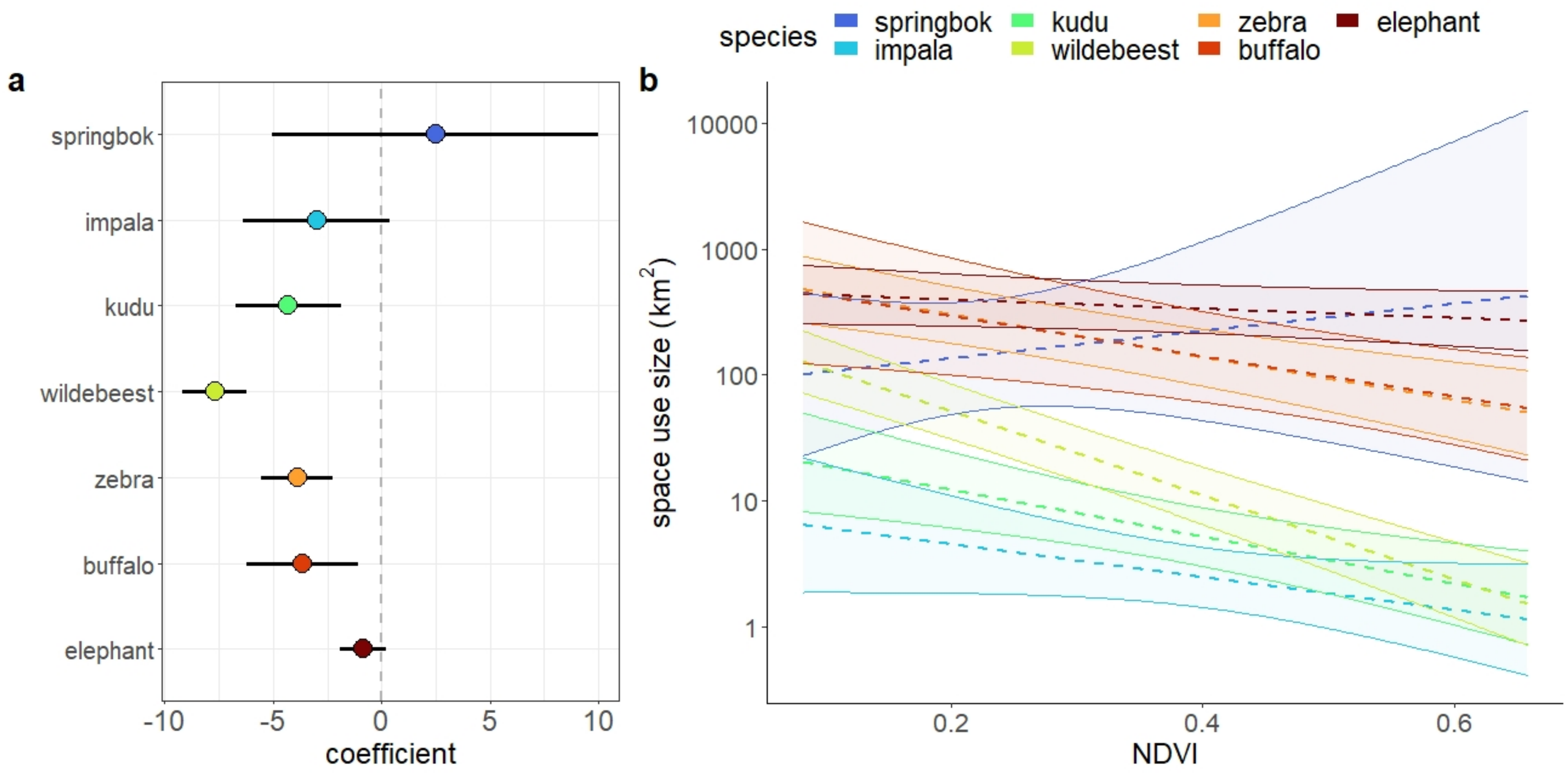

species
springbok
impala
kudu
wildebeest
zebra
buffalo
elephant
a
springbok
impala
kudu
wildebeest
zebra
buffalo
elephant
-10
-5
0
5
10
coefficient
b
10000
1000
100
10
1
space use size (km²)
0.2
0.4
0.6
NDVI

Figure S7

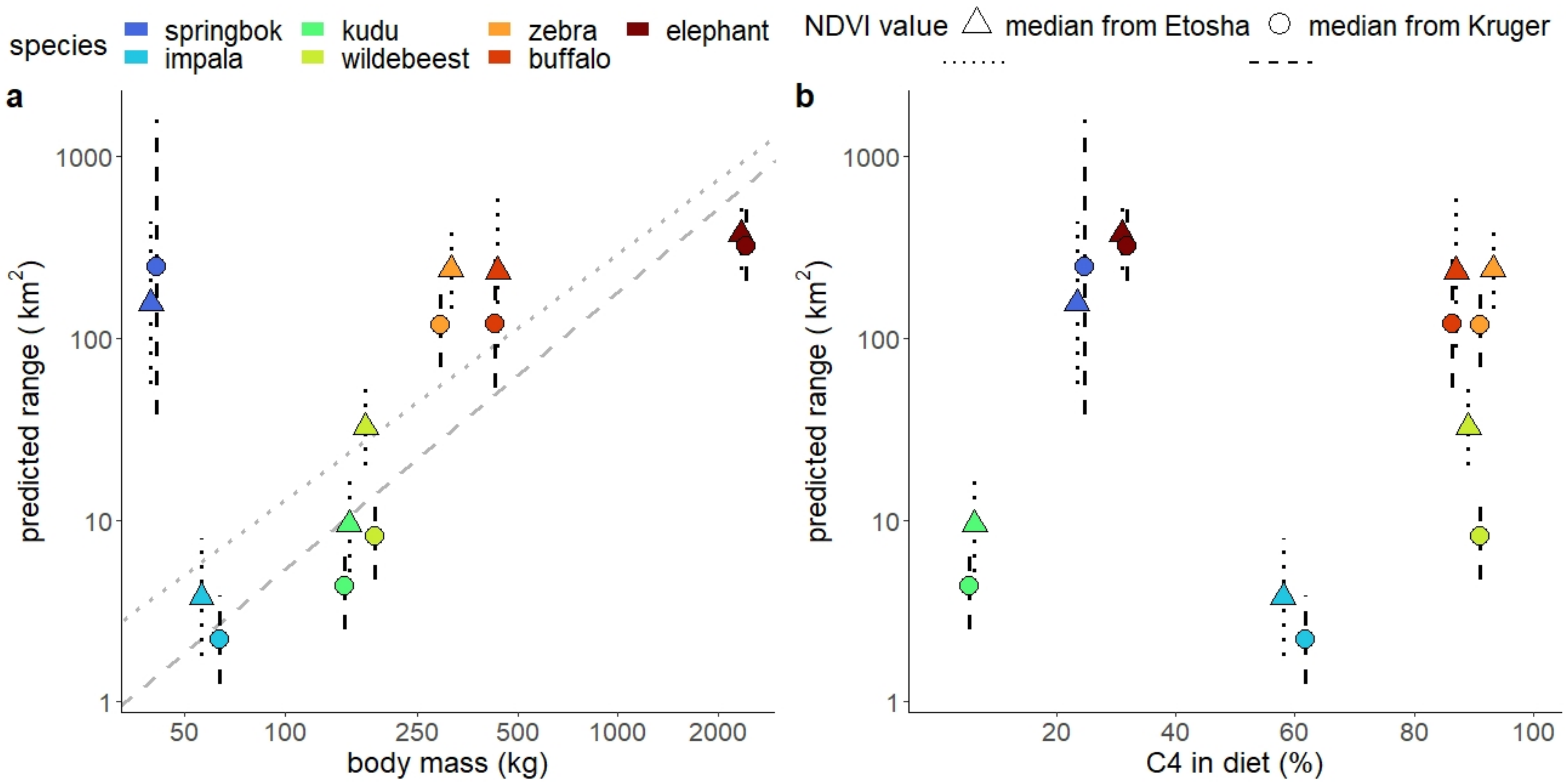
species
springbok
impala
kudu
wildebeest
zebra
buffalo
elephant
NDVI value
median from Etosha
median from Kruger
a
predicted range (km²)
1000
100
10
1
50
100
250
500
1000
2000
body mass (kg)
b
predicted range (km²)
1000
100
10
1
20
40
60
80
100
C4 in diet (%)

Figure S8

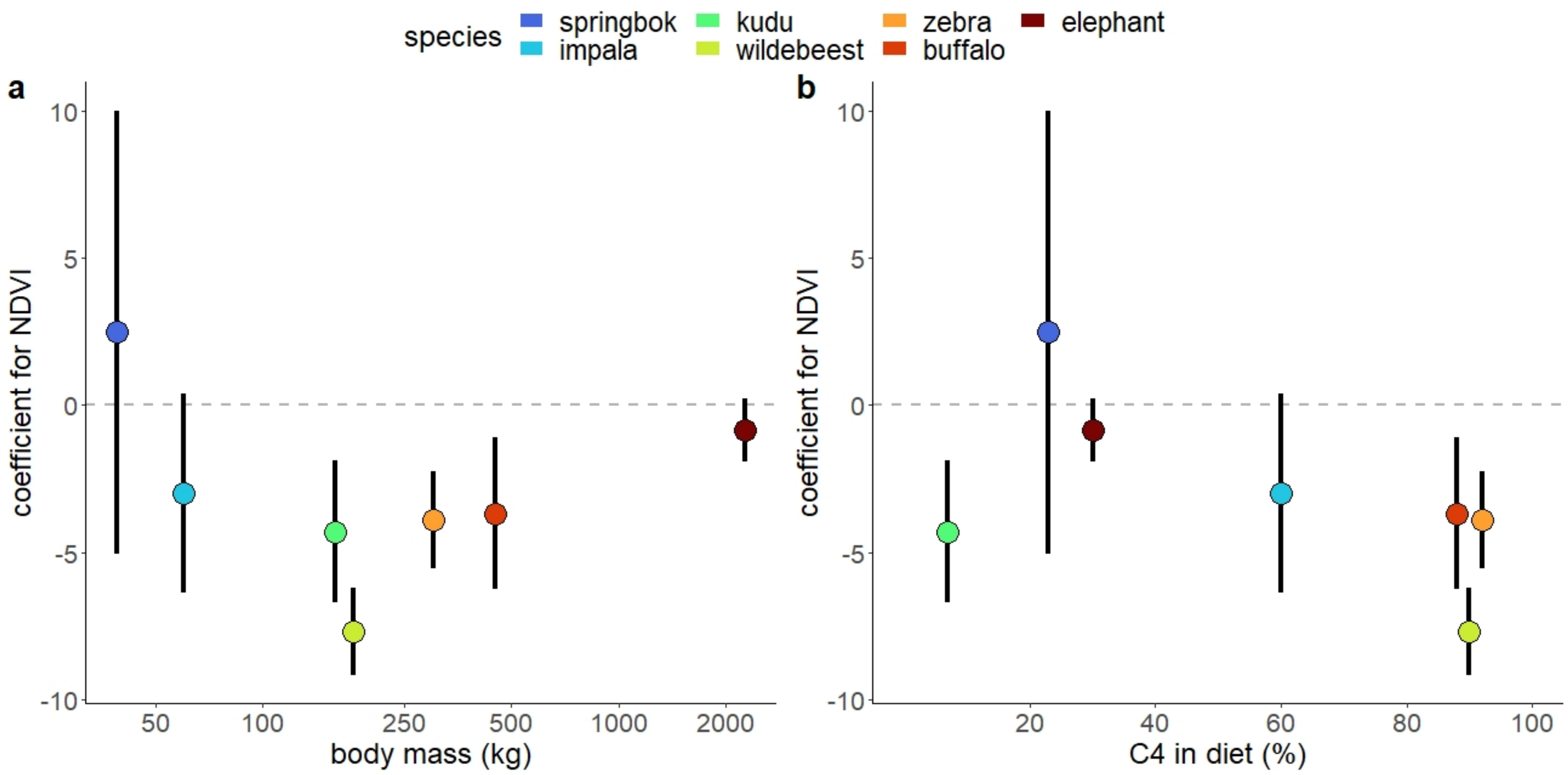
species
springbok
impala
kudu
wildebeest
zebra
buffalo
elephant
a
b
coefficient for NDVI
10
5
0
-5
-10
50
100
250
500
1000
2000
body mass (kg)
20
40
60
80
100
C4 in diet (%)

Figure S9

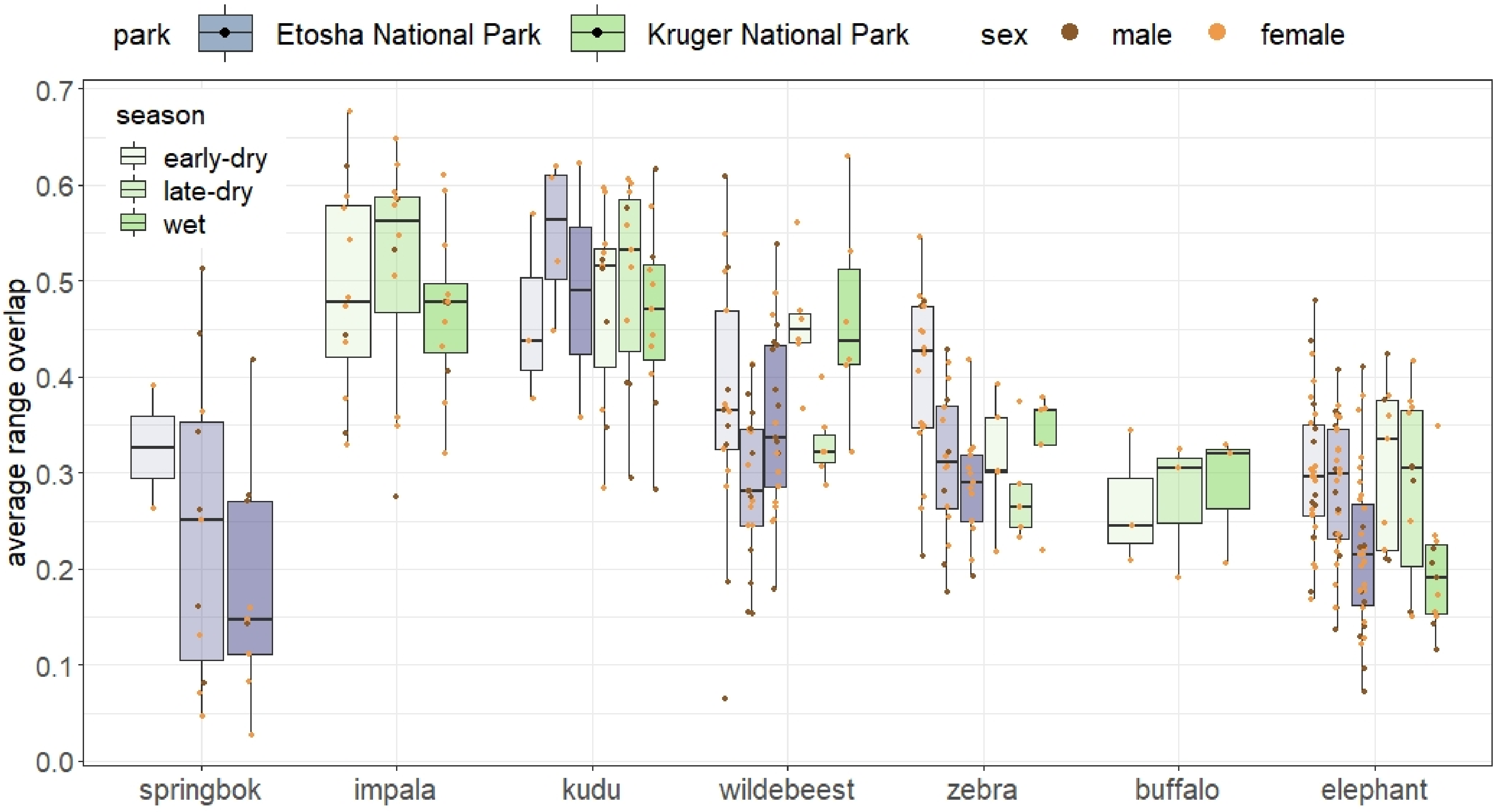

park
Etosha National Park
Kruger National Park
sex
male
female
season
early-dry
late-dry
wet
average range overlap
0.7
0.6
0.5
0.4
0.3
0.2
0.1
0.0
springbok
impala
kudu
wildebeest
zebra
buffalo
elephant

Figure S10

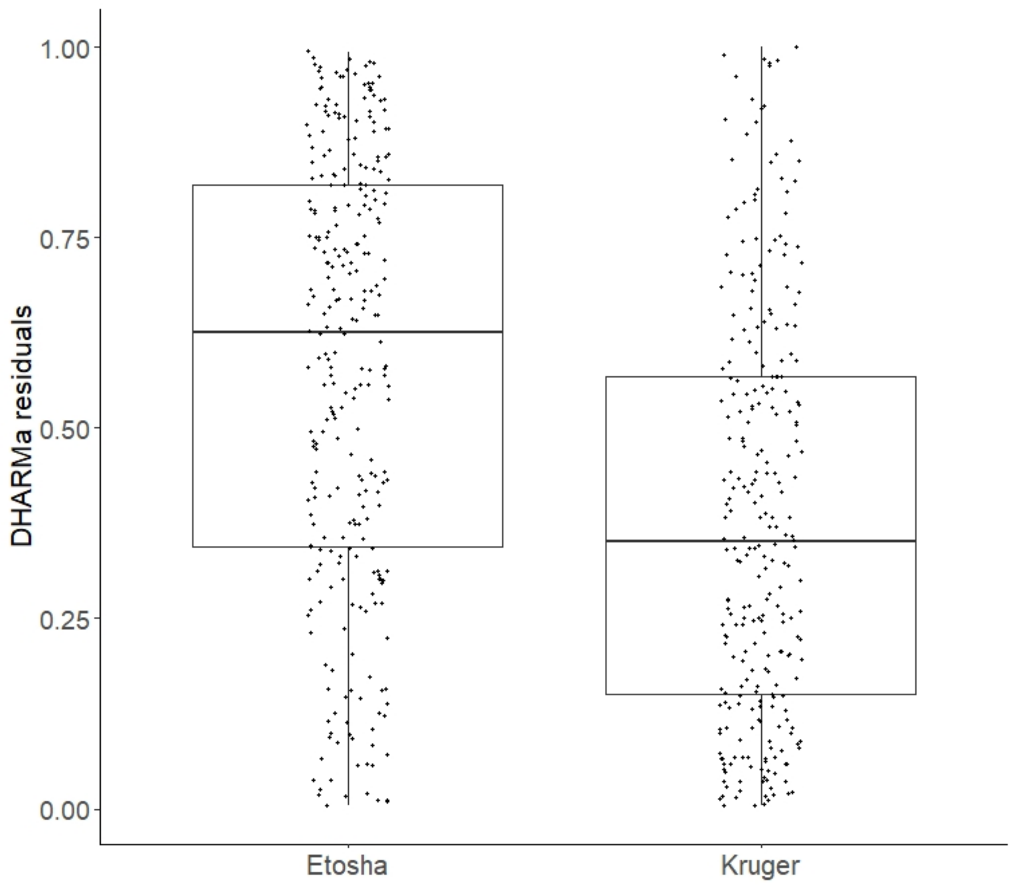

Supplement: Supplementary file 3 — Supplementary Material 3 [file 40462_2023_385_MOESM3_ESM.pdf]
